# Quisinostat is a brain-penetrant radiosensitizer in glioblastoma

**DOI:** 10.1172/jci.insight.167081

**Published:** 2023-11-22

**Authors:** Costanza Lo Cascio, Tigran Margaryan, Ernesto Luna-Melendez, James B. McNamara, Connor I. White, William Knight, Saisrinidhi Ganta, Zorana Opachich, Claudia Cantoni, Wonsuk Yoo, Nader Sanai, Artak Tovmasyan, Shwetal Mehta

**Affiliations:** 1 Ivy Brain Tumor Center and; 2 Department of Translational Neuroscience, Barrow Neurological Institute, St. Joseph’s Hospital and Medical Center, Phoenix, Arizona, USA.

**Keywords:** Oncology, Therapeutics, Brain cancer, Drug therapy, Radiation therapy

## Abstract

Histone deacetylase (HDAC) inhibitors have garnered considerable interest for the treatment of adult and pediatric malignant brain tumors. However, owing to their broad-spectrum nature and inability to effectively penetrate the blood-brain barrier, HDAC inhibitors have failed to provide substantial clinical benefit to patients with glioblastoma (GBM) to date. Moreover, global inhibition of HDACs results in widespread toxicity, highlighting the need for selective isoform targeting. Although no isoform-specific HDAC inhibitors are currently available, the second-generation hydroxamic acid–based HDAC inhibitor quisinostat possesses subnanomolar specificity for class I HDAC isoforms, particularly HDAC1 and HDAC2. It has been shown that HDAC1 is the essential HDAC in GBM. This study analyzed the neuropharmacokinetic, pharmacodynamic, and radiation-sensitizing properties of quisinostat in preclinical models of GBM. It was found that quisinostat is a well-tolerated and brain-penetrant molecule that extended survival when administered in combination with radiation in vivo. The pharmacokinetic-pharmacodynamic-efficacy relationship was established by correlating free drug concentrations and evidence of target modulation in the brain with survival benefit. Together, these data provide a strong rationale for clinical development of quisinostat as a radiosensitizer for the treatment of GBM.

## Graphical abstract

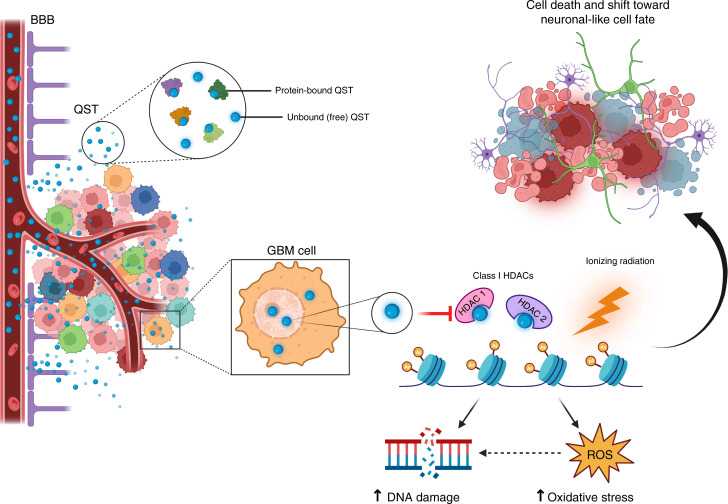

## Introduction

Histone deacetylase (HDAC) inhibitors (HDACis) are a successful example of epigenetic therapy, with 5 inhibitors currently approved by the US Food and Drug Administration for treatment of different hematological malignancies and a growing number of agents currently in different stages of clinical testing for a variety of cancers (1). Over the past 15 years, numerous HDACis have been studied preclinically in neuro-oncology, and 3 HDACis (vorinostat, romidepsin, and panobinostat) have been tested in clinical trials for patients with primary and recurrent glioblastoma (GBM) (2). Unfortunately, none has substantially prolonged survival in patients with GBM. The disappointing clinical results of HDACis in GBM treatment are attributable to inadequate disease modeling at the preclinical level, poor blood-brain barrier (BBB) penetration, and limited central nervous system (CNS) pharmacokinetic profiling (2–4).

HDACis clinically tested for GBM thus far have been broad-spectrum HDACis (pan-HDACis), which target multiple human HDAC isoforms (2). Because HDACs retain essential functions across different tissues, pan-HDACis (e.g., the hydroxamic acid panobinostat) can be associated with serious adverse events (5–7). Thus, HDACi-induced toxicities restrict the therapeutic window for treating CNS malignancies. However, improved drug target selectivity typically leads to a superior safety profile, and this may hold true for HDACis as well (8, 9).

Isoform selectivity of HDACis is an important consideration given that not all HDAC enzymes are equally expressed in GBM, and the specific roles of individual HDAC isoforms in these tumors are poorly understood (10). We recently uncovered the functional importance of HDAC1, an HDAC isoform whose expression increases with brain tumor grade and is correlated with decreased survival, in GBM (11). We found that HDAC1 function is essential for the survival of glioma stem cells (GSCs) and that its loss is not compensated for by its paralogue HDAC2 or other HDACs. Importantly, the loss of HDAC1 alone prolonged survival in vivo, providing a rationale for developing isoform-selective HDACis for GBM treatment (11).

Although no HDAC1-selective agents are currently available, quisinostat (QST) (JNJ-26481585) is a second-generation HDACi that is highly selective toward class I HDACs and harbors marked potency toward HDAC1 (half-maximal inhibitory concentration [IC_50_]: 0.1 nM) (8). QST has shown potent antitumor activity in preclinical models of different cancers and has been studied in phase I/II clinical trials for ovarian and hematological malignancies (12, 13). However, the survival benefits of QST treatment in orthotopic settings for brain tumors remain controversial (GL261, sonic hedgehog medulloblastoma, diffuse intrinsic pontine glioma [DIPG]) (14–16). Notably, these studies lacked pharmacokinetic (PK) and pharmacodynamic (PD) data and/or were conducted in flank tumor models.

Here, we report detailed CNS and brain tumor PK, PD, and radiation-sensitizing properties of QST in preclinical models of GBM. We found that QST inhibits the growth of multiple GSC lines and induces histone hyperacetylation, DNA damage, cell death, and cell cycle arrest. We demonstrate that QST is a brain-penetrant molecule that can extend survival of an orthotopic patient-derived xenograft model of GBM when combined with ionizing radiation (IR) therapy. Our results identify QST as a potent radiosensitizer, providing a rationale for clinical development of QST in combination with IR for GBM treatment.

## Results

### Cytotoxicity and induction of stable global hyperacetylation in patient-derived GSCs treated with QST.

**Figure 1:**
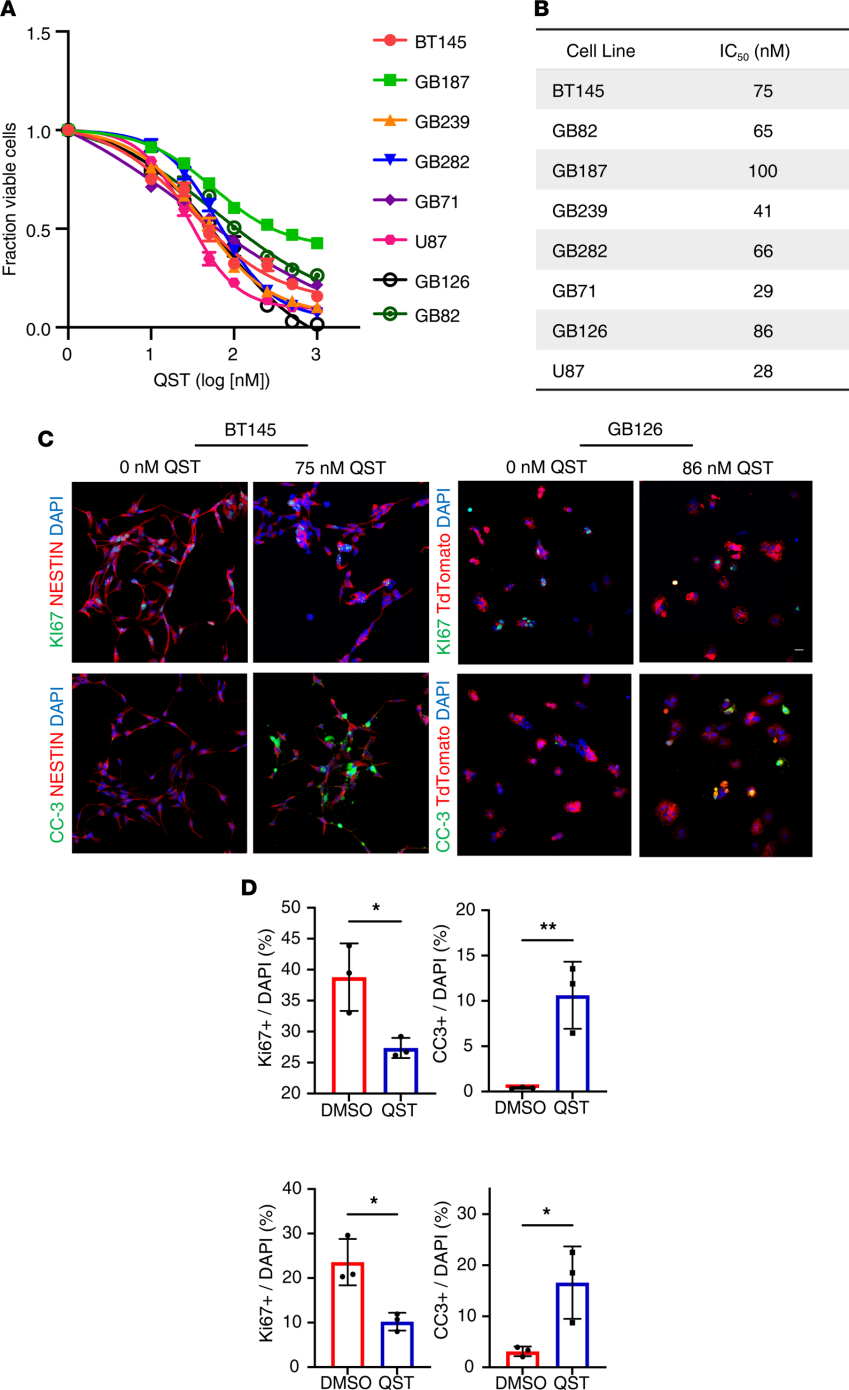
QST exhibits low nanomolar efficacy against human GSC cultures. (**A**) Dose-response curves with QST (10–1000 nM). Cell viability was measured across 7 patient-derived GSCs and 1 serum-grown long-term glioma line (U87) 3–5 days after treatment with QST. (**B**) Table illustrating the IC_50_ of QST for each cell line tested. (**C**) Immunofluorescent staining of GSC lines BT145 (left) and GB126 (right) 72 hours after treatment with QST at the IC_50_ concentrations. Control and drug-treated cells were stained for Ki67 and cleaved caspase-3 (CC3) to assess cell proliferation and cell death, respectively. Original magnification, ×20. Scale bar: 20 μm. (**D**) Quantification of Ki67-positive and CC3-positive cells (*n* = 3 mice per cell line). The dots or squares indicate values, the bar indicates the mean value, and the error bars indicate SEM. *P < 0.05, **P < 0.01 by unpaired, 2-tailed Student’s *t* test. For each cell line, the data are compiled from at least 3 independent experiments.

**Figure 2:**
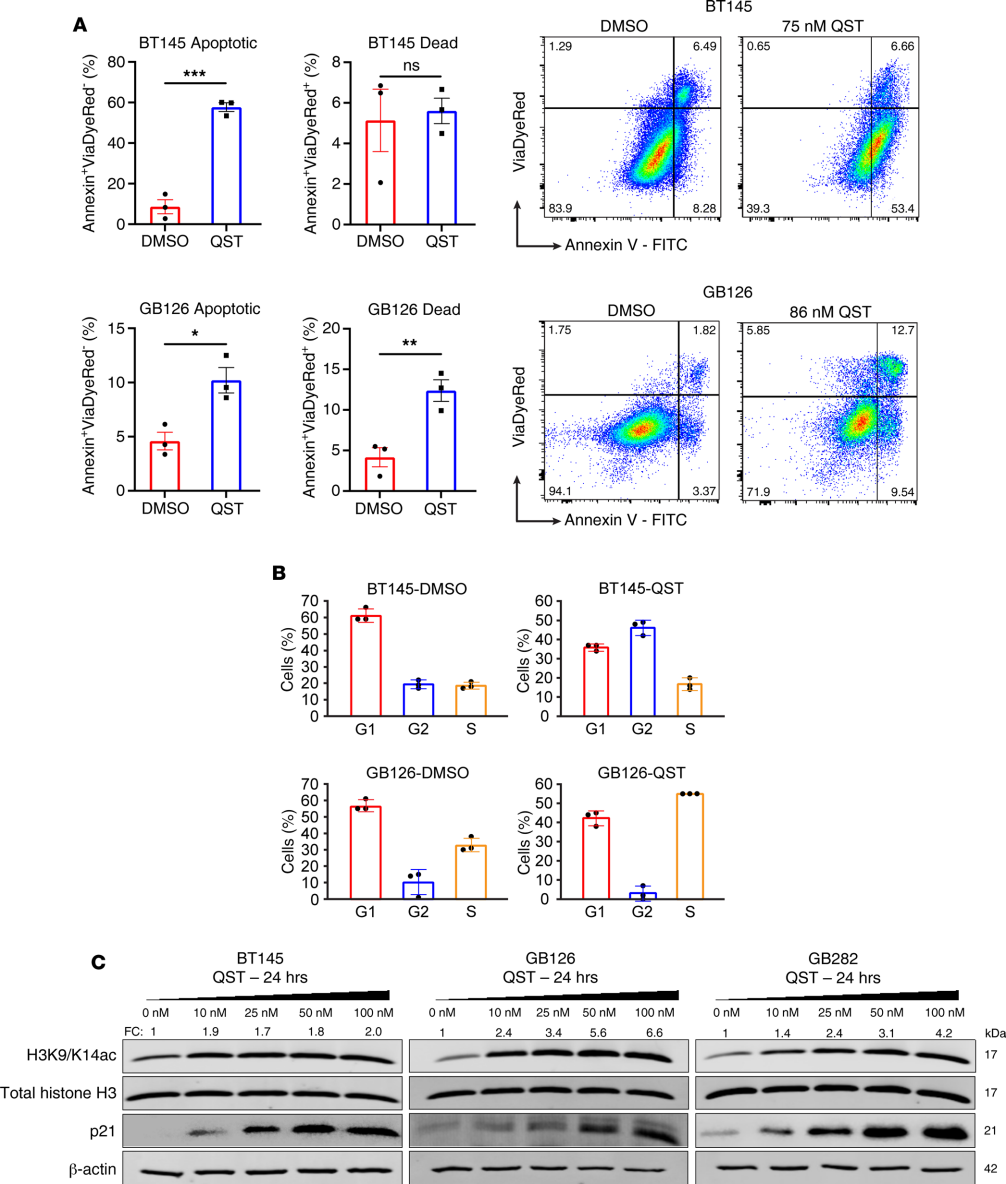
Short-term treatment with QST induces apoptosis and cell cycle arrest in human GSC cultures. (**A**) Flow cytometry analysis of apoptosis through annexin V staining in BT145 (top) and GB126 (bottom). Representative flow cytometry dot plots of cells stained for annexin and ViaDye Red counterstain in DMSO- and QST-treated cells. The dots or squares indicate values, the bar indicates the mean value, and the error bars indicate SEM. (**B**) Mean proportion of cells in each phase of the cell cycle in BT145 and GB126 cells 24 hours after treatment with DMSO or QST, assessed by propidium iodide staining through flow cytometry (*n* = 3 mice per cell line). BT145-DMSO: G_1_, 61%; S, 19%; G_2_, 18%. BT145-QST: G_1_, 36%; S, 17%; G_2_, 46%. GB126-DMSO: G_1_, 57%; S, 33%; G_2_, 10%. GB126-QST: G_1_, 42%; S, 55%; G_2_, 3%. (**C**) Representative immunoblots showing dose-dependent increase in histone H3 acetylation and p21 levels in GSC lines after 24-hour treatment with QST. Fold change (FC) values are indicated above H3K9/14ac bands to indicate changes in acetylated histone H3 relative to DMSO-treated cells. *P < 0.05, **P < 0.01 by unpaired, 2-tailed Student’s *t* test. For each cell line, the data are compiled from at least 3 independent experiments.

QST (JNJ-26481585) is a second-generation hydroxamic acid harboring remarkable selectivity toward class I HDACs, with a biochemical IC_50_ of 0.1 nM for HDAC1 (13). QST has demonstrated in vitro efficacy across multiple human cell lines derived from aggressive pediatric brain tumors (15, 16), but not GBM cell lines. To determine the cytotoxic effects of QST, we performed a dose-titration cell viability assay in 7 patient-derived GSC lines (BT145, GB187, GB239, GB282, GB71, GB82, and GB126) and 1 long-term serum-grown human GBM cell line (U87). Because GBMs display a high degree of intratumoral heterogeneity, we used GSCs derived from both primary and recurrent GBMs that harbored distinct genetic mutations or aberrations, growth rates, *MGMT* promoter methylation status, and gene expression profiles (Supplemental Figure 1; supplemental material available online with this article; https://doi.org/10.1172/jci.insight.167081DS1). Cell lines were treated with various concentrations of QST (10–1000 nM), and cell viability was measured 3–5 days later (Figure 1, A and B). The cellular IC_50_ for QST in all lines was in the low nanomolar range (50–100 nM), demonstrating greater potency (<1 μM) than other pan-HDACis (valproic acid, trichostatin A, vorinostat, entinostat) tested on GBM cells in other studies (17–22). Treatment with QST at the IC_50_ induced significant inhibition of proliferation (Ki67) and an increase in programmed cell death (cleaved caspase 3) in 2 different GSC lines (Figure 1, C and D). Additional analyses confirmed that QST induces apoptosis after short-term treatment through increased staining of the apoptotic marker annexin V (Figure 2A) and cell cycle arrest (in G_2_- or S-phase; Figure 2B and Supplemental Figure 1B). These results indicate a cytotoxic effect of QST on GSC cultures.

We next investigated the cellular effects of QST on histone acetylation dynamics in GSCs. Immunoblot analysis of lysates from 3 independent GSC lines (BT145, GB126, and GB282) treated with increasing concentrations of QST (10–100 nM) revealed a significant dose-dependent increase in histone H3 acetylation at lysines 9 and 14 (H3K9/14ac), indicative of target engagement, given that histone acetylation is primarily regulated by HDAC1 and HDAC2 (Figure 2C) (23). We observed a dose-dependent increase in the expression of p21, a tumor suppressor protein and a key negative regulator of the cell cycle (Figure 2C). These results suggest that QST induces global changes in histone hyperacetylation, increases chromatin accessibility, and promotes cell cycle arrest in GSCs.

### QST and DNA damage.

**Figure 3:**
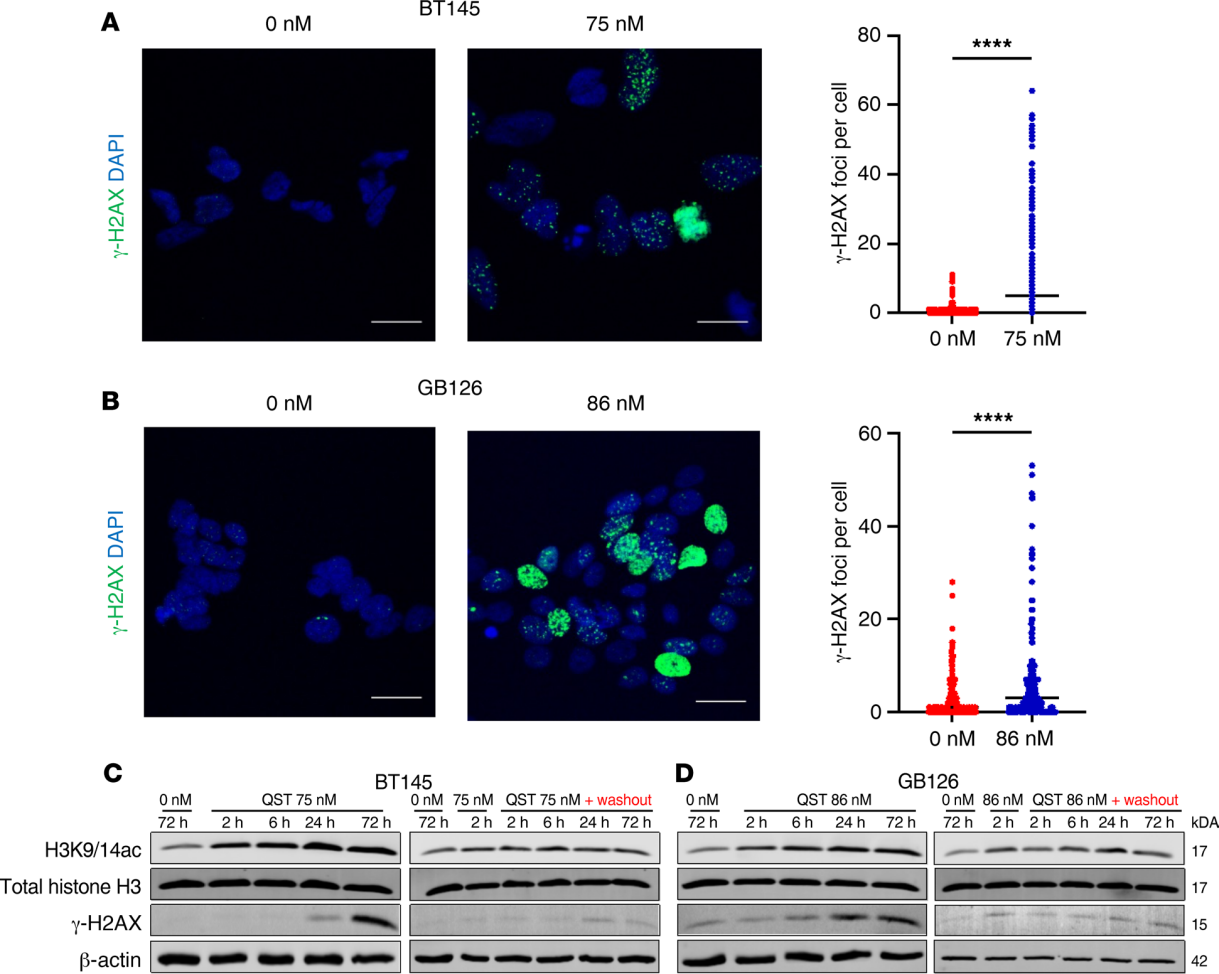
QST sensitizes GSCs to IR. Immunofluorescent staining of BT145 (**A**) and GB126 (**B**) showing an increase in γ-H2AX foci 72 hours after treatment in QST-treated cells but not in DMSO-treated cells. The mean number of γ-H2AX foci quantified in each cell per treatment condition is shown to the right for BT145 and GB126. Representative immunoblots demonstrating that QST treatment in BT145 (**C**) and GB126 (**D**) results in accumulation of γ-H2AX over time (left) and but not after drug washout (right). For each cell line, the data are compiled from at least 3 independent experiments. Original magnification, ×63. Scale bars: 20 μm. ****P < 0.0001 by unpaired, 2-tailed Student’s *t* test.

Previous studies have demonstrated that several HDACis can act as DNA-damaging agents in malignant cells (24–26). To test the DNA-damaging effects of QST in GBM cells, we treated 2 independent GSC lines with QST for 72 hours at the IC_50_ and analyzed changes in the levels of phosphorylated histone H2AX (γ-H2AX), a marker for DNA double-strand breaks (DSBs) (27). We found that there were significantly more γ-H2AX foci per cell in both cell lines after treatment compared with dimethylsulfoxide-treated (DMSO-treated) controls (Figure 3, A and B).

We next sought to understand the temporal dynamics of QST-induced DNA DSBs in GSCs. We treated 2 different GSC lines at their respective IC_50_ values for QST (75 nM for BT145 and 86 nM for GB126) and used immunoblotting to observe changes in γ-H2AX levels at 2, 6, 24, and 72 hours after treatment with the drug (Figure 3, C and D). We observed that histone H3 hyperacetylation increased over time after continuous exposure to QST, whereas γ-H2AX accumulated more gradually, with levels peaking at 72 hours after treatment in both cell lines. To characterize the reversibility of the expected PD effect, we performed cell washout experiments at different time points after drug removal. To determine whether histone H3 hyperacetylation and DNA damage persisted after acute exposure to QST, we replaced drug-spiked media with drug-free media 2 hours after treatment. Washout experiments found that QST target engagement (assessed through changes in histone H3K9/14 acetylation relative to DMSO-treated cells) persisted after drug removal from the media, albeit not to the levels achieved during persistent drug exposure. Moreover, in both cell lines, we found that γ-H2AX levels did not dramatically increase after drug washout. These results indicate that QST-induced inhibition of class I HDAC activity is relatively stable (24–72 hours) and capable of inducing a prolonged PD effect (histone acetylation) in GSCs, even after short-term drug incubation. The lack of significant accumulation in DNA DSBs suggests that continuous or persistent drug exposure is necessary for QST to induce substantial DNA damage in GSCs. To our knowledge, this is the first report to indicate that QST can act as a potent DNA-damaging agent in cancer cells in vitro.

### Kinetics of intracellular uptake of QST in GSCs.

To further understand the kinetics of drug-target engagement in GSCs, we harvested media and BT145 cells at 0, 2, 6, 10, and 24 hours with or without removal of QST (75 nM). At each time point, we measured the intracellular drug levels and concentrations in the cell media. With continuous drug exposure (no washout), the intracellular levels of QST in BT145 increased throughout the incubation period, reaching equilibrium by 10 hours (Supplemental Figure 2A). However, levels of QST decreased in cell media over time, suggesting that the drug may be unstable in GSC culture media. By contrast, in washout experiments (performed 2 hours after spiking cell media with the drug), minimal QST levels were measured intracellularly after drug removal at all time points (Supplemental Figure 2B). Our results indicate that the low intracellular drug levels after washout (<10 nM; Supplemental Figure 2B) are sufficient to inhibit HDAC activity, although inhibition is short-lived compared with that achieved with continuous drug exposure (Figure 3C).

### QST treatment and sensitization of GSCs to IR in vitro.

**Figure 4:**
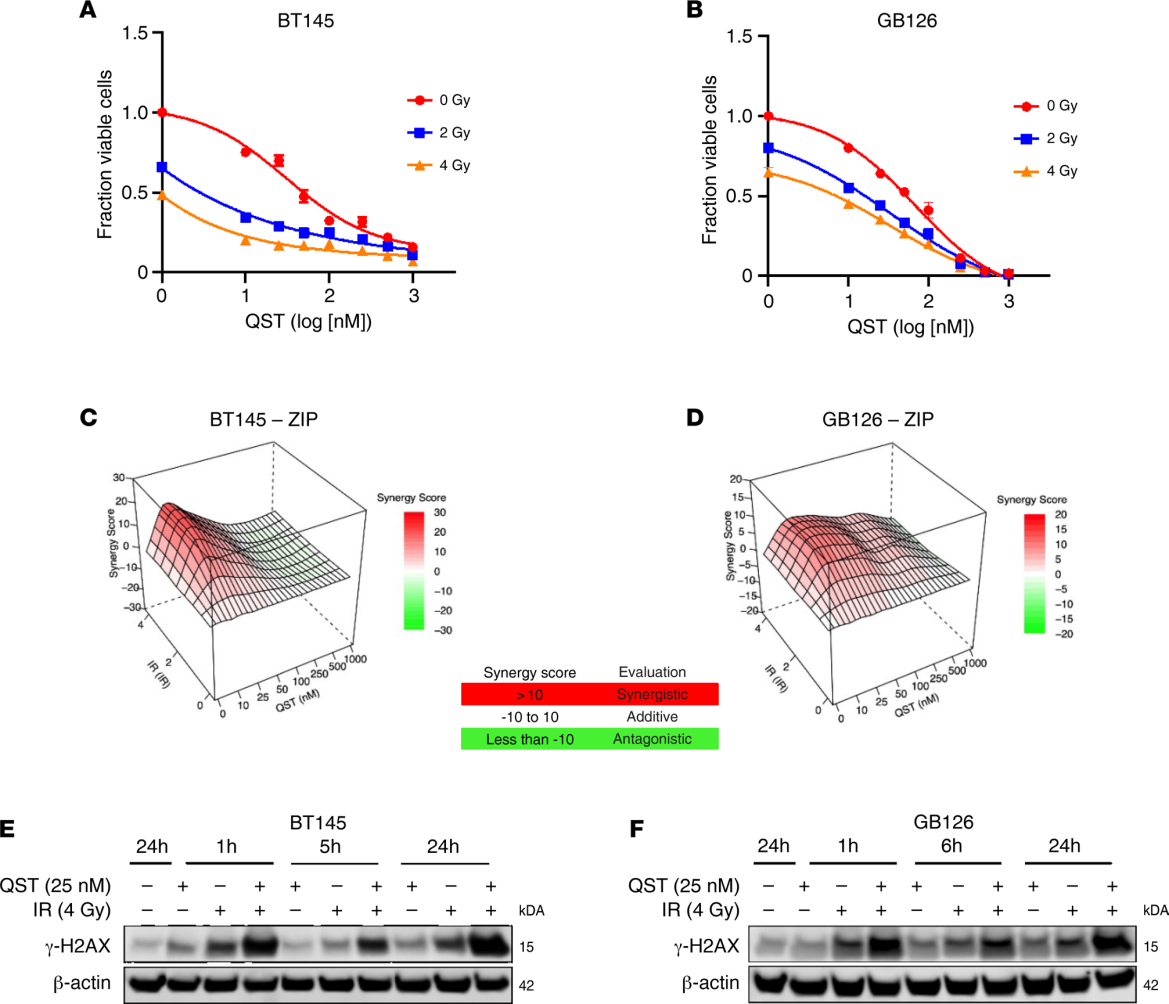
QST radiosensitizes GSCs in vitro. Dose-response curves combining QST and IR treatment in BT145 (**A**) and GB126 (**B**). Matrices illustrate the zero interaction potency synergy scores when combining QST with increasing doses of IR in BT145 (**C**) and GB126 (**D**). Representative immunoblots show protein levels of γ-H2AX in BT145 (**E**) and GB126 (**F**) 1, 6, and 24 hours after treatment with either QST alone (25 nM), IR alone (4 Gy), or both QST and IR (25 nM and 4 Gy, respectively). For each cell line, the data are compiled from at least 3 independent experiments.

There is preclinical evidence that HDACis may be effective in enhancing the sensitivity of tumor cells to IR therapy (24, 28–31). We hypothesized that the accumulation of DNA damage induced by QST, in combination with IR, may synergistically reduce GSC viability. To examine this hypothesis, we treated 2 cell lines (BT145 and GB126) with increasing nanomolar doses of QST (10–1000 nM) and increasing doses of IR (2–4 Gy) (Figure 4, A and B). Across both cell lines, combination treatment resulted in greater cytotoxicity than independent treatment with QST or IR (Figure 4, A and B), as measured through the CellTiter-Glo (Promega) luminescent cell viability assay. We then analyzed our combinatorial dose-response cell viability data using SynergyFinder, an application that assigns synergy scores using various major reference models (32). The zero interaction potency and Bliss and Loewe model synergy matrices indicated that the greatest synergy was attained when combining IR with the lowest doses of QST tested (10–25 nM) (Figure 4, C and D, and Supplemental Figure 3, A and B). We corroborated these results by performing the sulforhodamine B assay, which has comparable sensitivity to the standard clonogenic assay when assessing cytotoxicity (33). We found that low doses of QST and IR trended toward synergy in both cell lines tested (Supplemental Figure 3, C and D). Furthermore, immunoblotting analysis of BT145 and GB126 cells treated with 25 nM QST and/or 4 Gy of IR revealed that combinatorial treatment resulted in significantly higher levels of γ-H2AX than either treatment alone (Figure 4, E and F). Together, these data indicate low nanomolar doses of QST can enhance the sensitivity of GSCs to IR.

### Determination of optimal route of QST administration in vivo.

**Figure 5:**
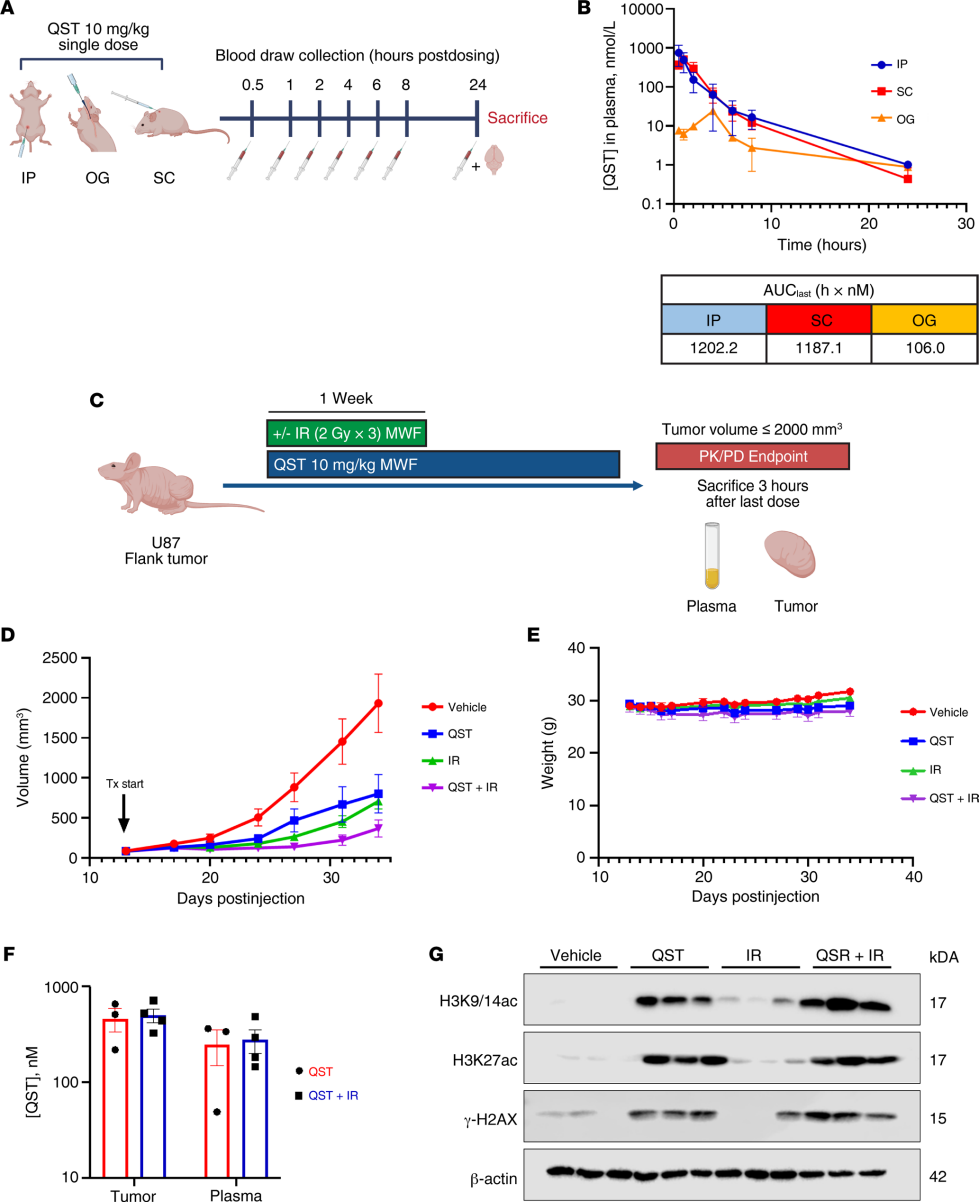
QST is effective in slowing tumor growth in a flank model of human GBM. (**A**) Schematic illustrating the experimental design. Athymic nude mice were treated with a single dose of QST (10 mg/kg) through IP injection, SC injection, or OG. Blood samples were collected at 0.5, 1, 2, 4, 6, 8, and 24 hours after dosing and analyzed by LC-MS/MS. (**B**) Total plasma concentration–time curve for QST administered through various routes. Values for AUC_last_ were calculated for each route to illustrate plasma QST exposure (bottom). Error bars indicate SEM. (**C**) Schematic illustrating the treatment regimen for mice with flank tumors. When the tumors reached a mean volume of 100 mm^3^, mice were randomized into 4 groups: vehicle, 10 mg/kg QST, IR alone (6 Gy), or combination treatment (6 Gy IR and 10 mg/kg QST) (*n* = 10 mice in each cohort). IR was given in fractionated doses (2 Gy MWF) only during the first week of treatment, with or without QST. Following completion of IR, mice in the monotherapy and combination cohorts continued to receive QST alone on MWF until the tumors reached the indicated volume threshold. (**D**) Weekly mean volume measurements of U87 flank tumors from mice treated with vehicle, QST, IR, or a combination of QST and IR (QST+IR) (*n* = 10 mice in each cohort). Error bars indicate SEM. (**E**) Mean weights of mice from each cohort throughout the study duration. Error bars indicate SEM. (**F**) Total levels of QST in plasma and flank tumors of mice treated with QST and QST+IR (*n* = 3 or 4 mice per cohort). Error bars indicate SEM. (**G**) Immunoblotting of protein lysate–derived homogenized flank tumors from each cohort (*n* = 3 mice per group). Membranes were probed for H3K9/14ac, H3K27ac, γ-H2AX, and β-actin. Differences were assessed using ordinary 1-way ANOVA with Dunnett’s multiple-comparison test.

Because the efficacy of QST in preclinical models of brain tumors remains controversial, and because previous studies employed various drug delivery methods, we sought to understand how different routes of administration affect the bioavailability of QST in mice. Three routes of administration were compared to determine the optimal dosing route to obtain the highest plasma exposure to QST over time. We treated athymic nude mice with a single dose of QST (10 mg/kg) through intraperitoneal (IP), subcutaneous (SC), or oral gavage (OG) delivery and collected blood at 0.5, 1, 2, 4, 6, 8, and 24 hours after dosing from individual mice for PK analysis (Figure 5A). Liquid chromatography with tandem mass spectrometry (LC-MS/MS) analysis revealed that, regardless of administration route, QST was systemically cleared within 24 hours of dosing (Figure 5B). QST injected IP and SC resulted in higher plasma exposure over time compared with OG delivery (AUC_last_ of 1202.2, 1187.1, and 106.0, respectively; Figure 5B). Therefore, IP delivery was the optimal route of administration to maximize the bioavailability of QST in athymic nude mice.

### QST inhibition of tumor growth in a flank model of human GBM.

We next examined whether QST treatment alone or in combination with IR would be effective in slowing tumor growth in a flank model of GBM (U87). Tumor-bearing mice receiving IR were treated with 2-Gy fractions on Monday, Wednesday, and Friday (MWF) 2 hours after dosing with either vehicle solution or QST (10 mg/kg), for a total dose of 6 Gy. Following completion of IR, mice continued to receive vehicle or QST until the tumors reached the maximum volume threshold (Figure 5C). Mice treated with QST monotherapy had significantly reduced tumor volume compared with vehicle-treated mice (Figure 5D). Combination treatment was more effective in reducing tumor growth than either QST or IR alone. The mean tumor volume in mice with combination treatment was approximately 4.5-fold less than that in vehicle-treated mice at study completion (Figure 5D). We found that QST, even in combination with IR, was well tolerated throughout the treatment study when dosed at 10 mg/kg IP MWF, with no significant loss in mean weights throughout the regimen (Figure 5E).

PK analyses of QST- and combination-treated mice demonstrated that mean total QST concentrations were slightly higher in flank tumors (approximately 433 nM) than in plasma samples (approximately 300 nM) (Figure 5F). There was no significant difference in the total levels of QST between the monotherapy and combination cohorts (Figure 5F). Immunoblotting confirmed a significant increase in histone H3 acetylation at lysines 9, 14, and 27 in mice treated with QST alone or in combination with IR (H3K9/14ac, H3K27ac; Figure 5G). Moreover, we observed that all QST-treated tumors expressed high levels of γ-H2AX, indicative of the presence of DSBs, compared with vehicle-treated controls (Figure 5G). These results suggest that 10 mg/kg dosing of QST is effective in reducing tumor burden and induces the intended PD effects in glioma cells, and they corroborate our previous in vitro findings that QST acts as a potent DNA-damaging agent.

### Interspecies differences in the stability of QST.

**Figure 6:**
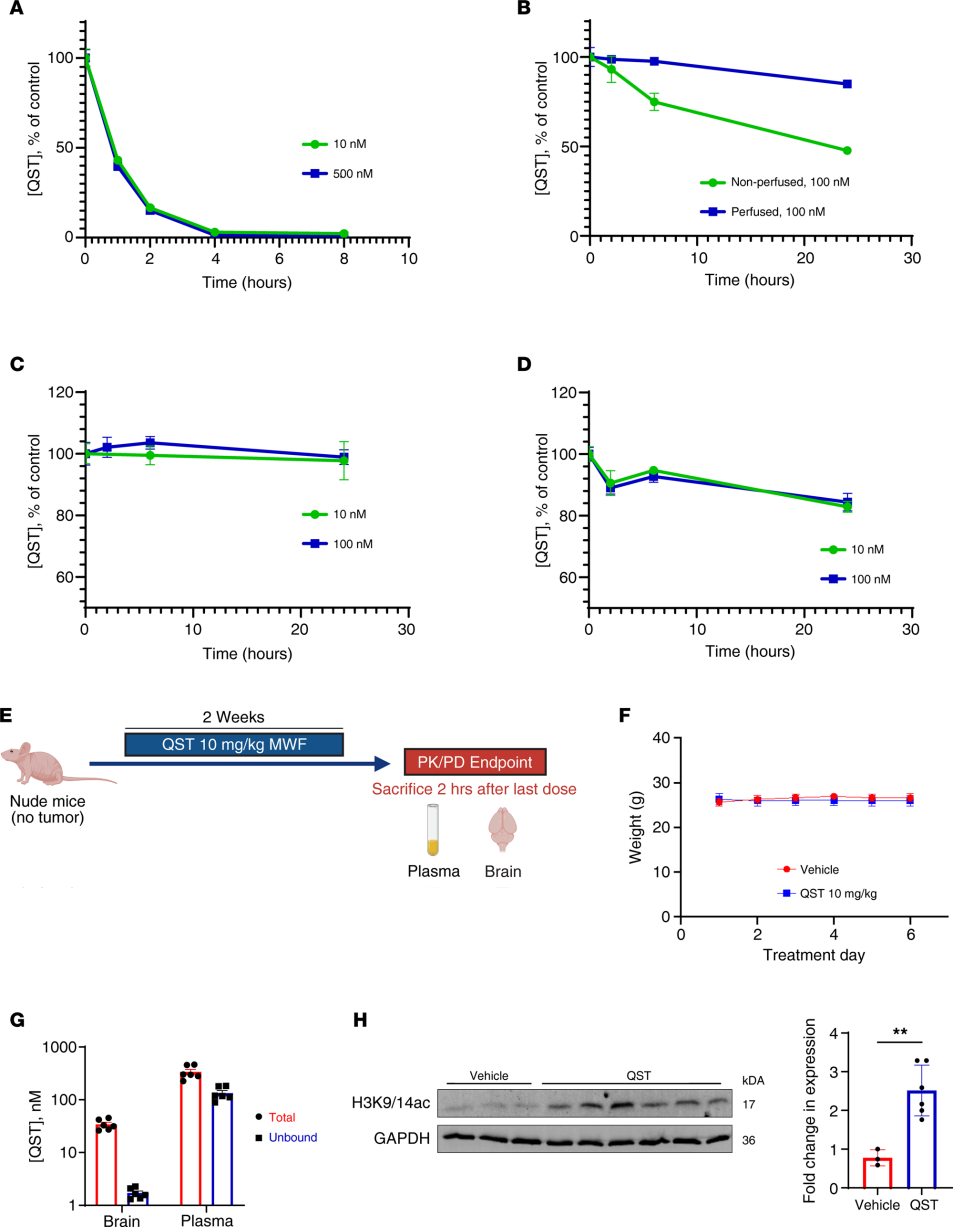
QST is a brain-penetrant HDACi. (**A**) Stability of QST (10 nM and 500 nM) in mouse plasma at 37°C. (**B**) Stability of QST in mouse nonperfused and perfused brain homogenate (1:7 weight/volume in PBS) at 37°C. (**C**) Stability of QST in human plasma at 37°C. (**D**) Stability of QST in human brain homogenate at 37°C. In **A**–**D**, values are the mean of triplicate measurements, and error bars indicate SEM. (**E**) Schematic illustrating the design of the treatment study in non–tumor-bearing athymic nude mice. Mice received treatment with vehicle or QST on MWF for 2 consecutive weeks and were euthanized 2 hours after the last dose of drug for PK and PD analyses. (**F**) Mean weights of mice in the vehicle- and QST-treated cohorts throughout the study duration (*n* = 10 mice per cohort). (**G**) Total and unbound levels of QST in normal brain tissue in QST-treated mice (*n* = 6 mice per cohort). (**H**) Immunoblotting of protein lysates derived from homogenized brains from each cohort (*n* = 3 mice for vehicle cohort, *n* = 6 mice for QST cohort). Membranes were probed for H3K9/14ac and GAPDH. Quantification of expression of H3K9/14ac (normalized to GAPDH) in QST- and vehicle-treated homogenized brain samples is shown to the right. **P < 0.01 by unpaired, 2-tailed Student’s *t* test. In **G** and **H**, circles and squares indicate values, bars indicate mean values, and error bars indicate SEM.

Hydroxamic acid–based compounds have been reported to display poor stability and high plasma clearance due to the presence of arylesterases and carboxylesterases in rodent blood (34). Therefore, we measured the stability of QST in mouse plasma and brain to determine whether drug degradation occurred during our sample preparations and equilibrium dialyses for the determination of unbound drug level (performed at 37°C). We found that QST exhibited instability in mouse plasma during an 8-hour incubation time, with a half-life of approximately 1 hour (Figure 6A). QST degradation was also observed in mouse brain homogenate (Figure 6B). However, perfusion of mice before brain collection prevented rapid drug degradation in the brain matrix. This indicates that QST instability in the brain is likely related to enzymes that are present in the mouse plasma. Degradation of QST can also be constrained if plasma and brain samples are processed at 4°C (Supplemental Figure 4A). All subsequent sample preparations were therefore performed on ice-cold baths to avoid degradation of QST in our PK analyses. Importantly, we demonstrate that the stability of QST can also be prolonged in the presence of the carboxylesterase inhibitor bis(*p*-nitrophenyl) phosphate (BNPP) (Supplemental Figure 6B). Hence, dialysis of mouse plasma and brain samples was performed in the presence of BNPP to maximize QST stability at 37°C. The drug was completely stable in human plasma and brain homogenate at 37°C (Figure 6, C and D). The observed stability is probably due to the absence of esterases in human matrices, which are responsible for degradation of QST (34). Because we employed QST in several in vitro studies (Figures 1–4), the stability of the molecule was also tested in the cell media used to culture GSCs (neurobasal medium; see Methods). We demonstrate that approximately 70% of QST stays intact in the cell media over a 24-hour incubation period at 37°C (Supplemental Figure 4C). These results agree with the data obtained in the intracellular uptake experiments illustrated in Supplemental Figure 2A.

### PK and PD of QST in normal CNS.

Insufficient drug exposure in the brain is a major hurdle in the treatment of brain tumors. Hence, we determined the PK profile of QST in the normal CNS by treating athymic nude mice with 10 mg/kg QST on a MWF schedule for 2 weeks. On the last treatment day, blood and intact brains were harvested 2 hours after dosing with QST (Figure 6E). QST was well tolerated throughout the short treatment study when dosed at 10 mg/kg IP MWF, and QST-treated mice exhibited no significant weight loss compared to vehicle-treated mice (Figure 6F). Each brain hemisphere was processed separately to perform matched PK and PD analyses from the same animal. We found that, although the mean unbound levels of QST were more than 70-fold lower in the brain than in the plasma, the pharmacologically active unbound drug concentration in the brain (approximately 1.7 nM) was more than 15 times higher than the biochemical IC_50_ for HDAC1 (0.1 nM) (Figure 6G). For PD analyses, we homogenized entire hemispheres to obtain whole-tissue protein lysates from each mouse and assessed changes in histone H3 acetylation levels using immunoblotting. We confirmed that the levels of H3K9/14 acetylation were significantly increased in the brain tissue of QST-treated mice (*n* = 6) compared with vehicle-treated mice (Figure 6H). Our results therefore indicate that QST is a brain-penetrant HDACi that exhibits clear on-target PD activity in normal CNS cells. We also established a direct correlation between PK and PD modulation in vivo, demonstrating that free unbound levels of QST in the brain can induce substantial histone H3 hyperacetylation (Figure 6H).

### QST as a radiosensitizer in an orthotopic patient-derived xenograft model of GBM.

**Figure 7:**
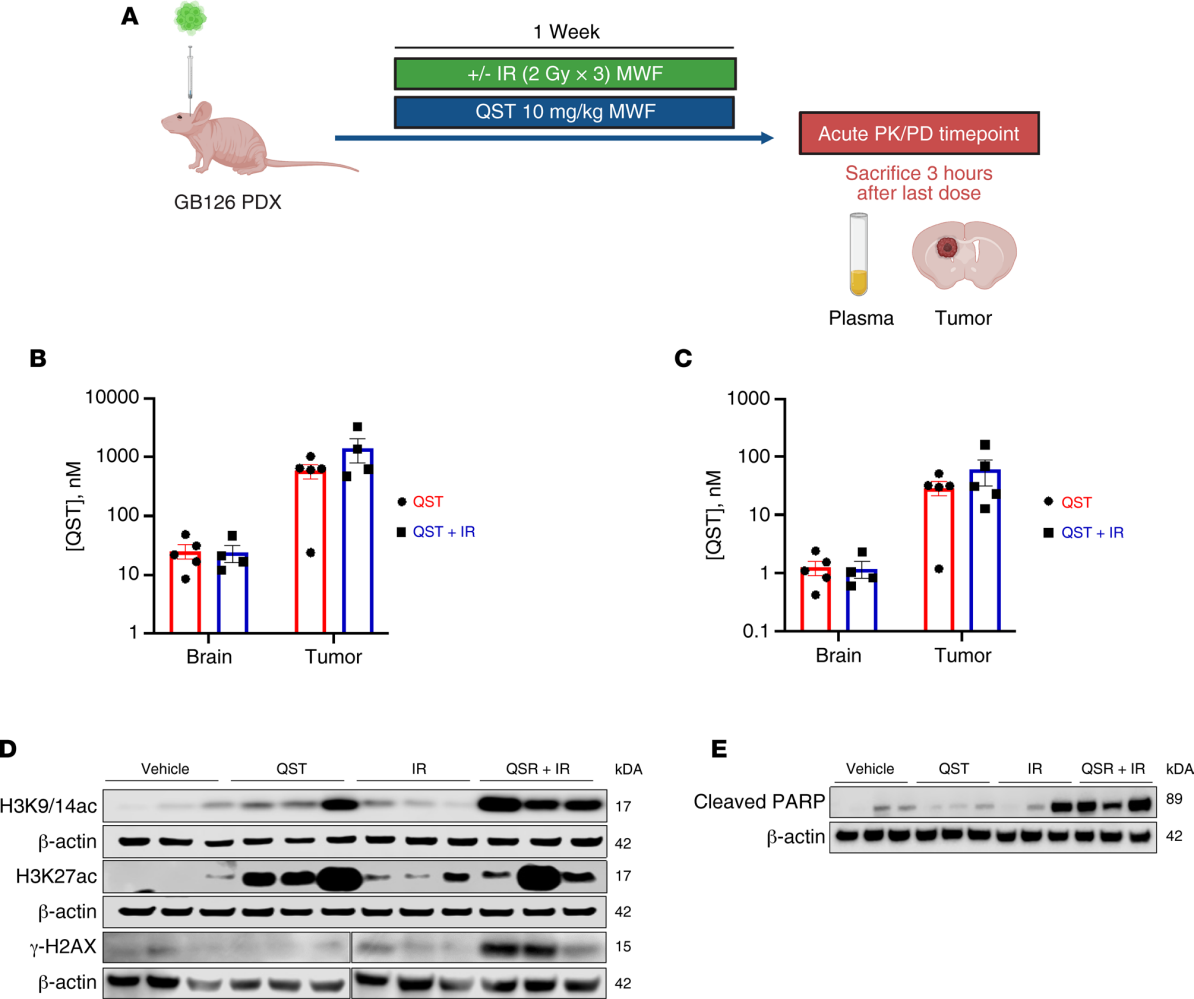
PK and PD analysis of QST in an orthotopic PDX model of GBM. (**A**) Schematic illustrating the experimental design of the treatment study in an orthotopic PDX model of GBM (GB126) to perform PK-PD analyses after short-term treatment (1 week) with QST and/or IR. Total (**B**) and unbound (**C**) levels of QST in tumor tissue and brain tissue contralateral to the tumor in mice treated with QST and QST+IR (*n* = 4 or 5 mice per cohort). Values are the mean of triplicate measurements. Circles and squares indicate values, bars indicate mean value, and error bars indicate SEM. (**D**) Immunoblotting of protein lysates derived from homogenized brain tumors from each cohort to assess changes in histone H3 acetylation and DNA damage (*n* = 3 mice per cohort). Membranes were probed for H3K9/14ac, H3K27ac, γ-H2AX, and β-actin. (**E**) Assessment of cell death as indicated by cleaved poly (ADP-ribose) polymerase (PARP) in homogenized brain tumors from each cohort (*n* = 3 mice per cohort).

To establish the PK-PD correlation and efficacy in an orthotopic patient-derived xenograft (PDX) model of GBM, we implanted GB126 cells in the brains of athymic nude mice and began treatment once the tumors started growing exponentially. Tumor-bearing mice were treated with QST (10 mg/kg), with or without IR, on a MWF schedule (Figure 7A). As described above, IR was delivered locally to the brain in 2-Gy fractions 2 hours after being dosed with vehicle solution or QST, for a cumulative delivery of 6 Gy. To assess acute PD effects and drug levels after short-term QST treatment (1 week, 3 doses total), 10 mice from each experimental cohort were sacrificed 3 hours after receiving the third dose of vehicle solution or QST (Figure 7A). Plasma, tumors, and brain tissue contralateral to the tumors were collected and processed for PK and PD analyses. As shown in Figure 7, B and C, total and unbound levels of QST accumulated in tumor tissue compared with contralateral brain tissue. However, unbound QST levels in the tumors were up to 600-fold higher than the biochemical IC_50_ for HDAC1 inhibition. Although QST levels in the contralateral brain tissue were significantly lower than those measured in the tumors, they were 12-fold higher than the biochemical IC_50_ of HDAC1, which ensures target inhibition in infiltrating tumor cells distant from the tumor core. No significant differences in drug accumulation were observed between the monotherapy and combination therapy cohorts. To assess PD modulation, we homogenized brain tumors to obtain whole-tissue protein lysates from each mouse and quantified changes in histone H3 acetylation levels using immunoblotting. We confirmed that, relative to vehicle-treated animals, the levels of H3K9/14 and H3K27 acetylation increased in monotherapy- and combination-treated mice (Figure 7D). Moreover, we detected elevated levels of γ-H2AX in combination-treated tumors, corroborating our previous findings that combining QST and IR results in high levels of DNA damage in GSCs in vitro (Figure 4, E and F). Detection of cleaved poly (ADP-ribose) polymerase (PARP) in tumor tissues also confirmed that combination treatment induced more cell death than monotherapy regimens (Figure 7E). Very low levels of cleaved PARP were detected in brain tissue from the contralateral hemisphere, indicating that tumor cells are more vulnerable to drug plus IR–induced cell killing in vivo relative to normal neural cells (Supplemental Figure 5A). In a dose response assay performed in vitro, we found that primary human astrocytes are equally sensitive to QST treatment (Supplemental Figure 5B). However, as mentioned above, this cytotoxic effect was not evident in normal brain tissue in vivo. These discordant results highlight that drug cytotoxicity in nontumorigenic cells should also be assessed in a physiologically relevant context (in this case, within the mouse brain).

**Figure 8:**
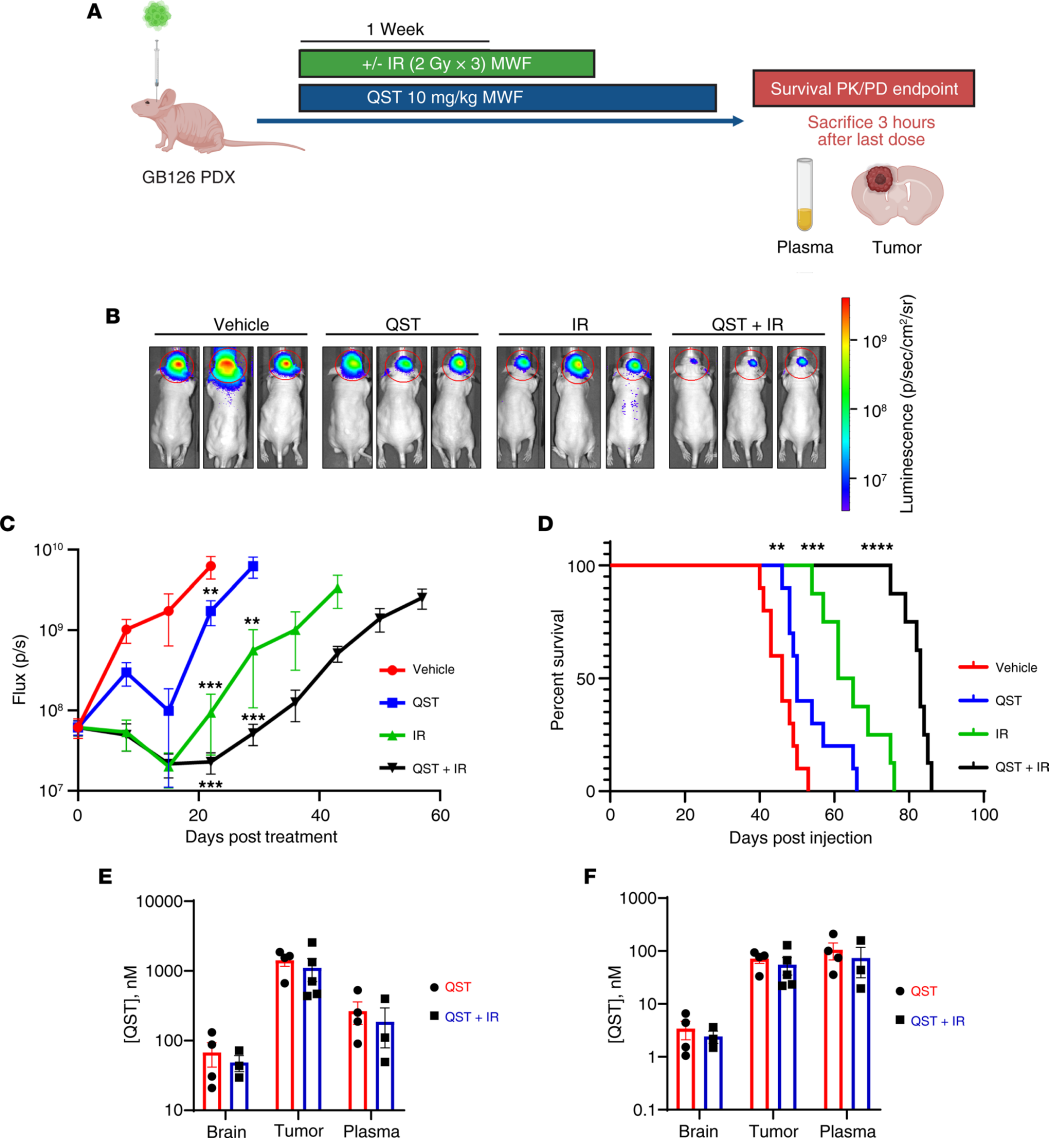
QST is a potent radiosensitizer in an orthotopic PDX model of GBM. (**A**) Schematic illustrating the experimental design of the treatment study in an orthotopic PDX model of GBM (GB126) to assess survival benefit across all treatment cohorts. Mice received IR only in the first week of treatment. Mice continued to receive QST treatment alone until they displayed neurological symptoms (the survival PK and PD endpoint). QST and IR were administered MWF. (**B**) Representative heatmap images of bioluminescence intensity across all treatment cohorts 22 days after treatment initiation. (**C**) Mean photon flux (p/s) measured through bioluminescence imaging across all cohorts throughout the entire duration of the treatment. Error bars indicate SEM. (**D**) Kaplan-Meier survival analysis of mice treated with vehicle, QST (10 mg/kg), IR (6 Gy), or QST+IR (6 Gy IR and 10 mg/kg QST) mice. Total (**E**) and unbound (**F**) levels of QST in tumor tissue and brain tissue contralateral to the tumor in mice treated with QST and QST+IR (*n* = 4 or 5 mice per cohort). Circles and squares indicate values, bars indicate mean values, and error bars indicate SEM. **P < 0.01, ***P < 0.001, ****P < 0.0001 by unpaired, 2-tailed Student’s *t* test (**C**) or Kaplan-Meier method with the Mantel-Cox log-rank test (**D**).

We additionally questioned whether QST, either alone or in combination with IR, could extend the survival of tumor-bearing mice. In this study, mice received the same treatments described above, but after completion of the 1-week IR regimen, mice continued to receive QST at 10 mg/kg or vehicle solution on MWF until study completion, as determined by large tumor burden and onset of neurological symptoms (Figure 8A). QST or QST+IR significantly reduced tumor burden compared with vehicle or IR-only cohorts (Figure 8, B and C). Although QST monotherapy slowed tumor growth, median survival for the QST-treated mice was only 4 days longer than for the vehicle-treated mice (*P* < 0.01), and IR alone increased median survival by 17 days (*P* < 0.001; Figure 8D). However, combining QST with IR led to a significant increase in median survival (37 days, *P* < 0.001) compared with the vehicle (Figure 8D). These data suggest that, although QST monotherapy produces a modest therapeutic benefit, combinatorial treatment with fractionated doses of IR reveals that QST acts a potent radiosensitizer that significantly prolongs survival in an orthotopic PDX model of human GBM compared with either monotherapy cohort (Figure 8D).

**Figure 9:**
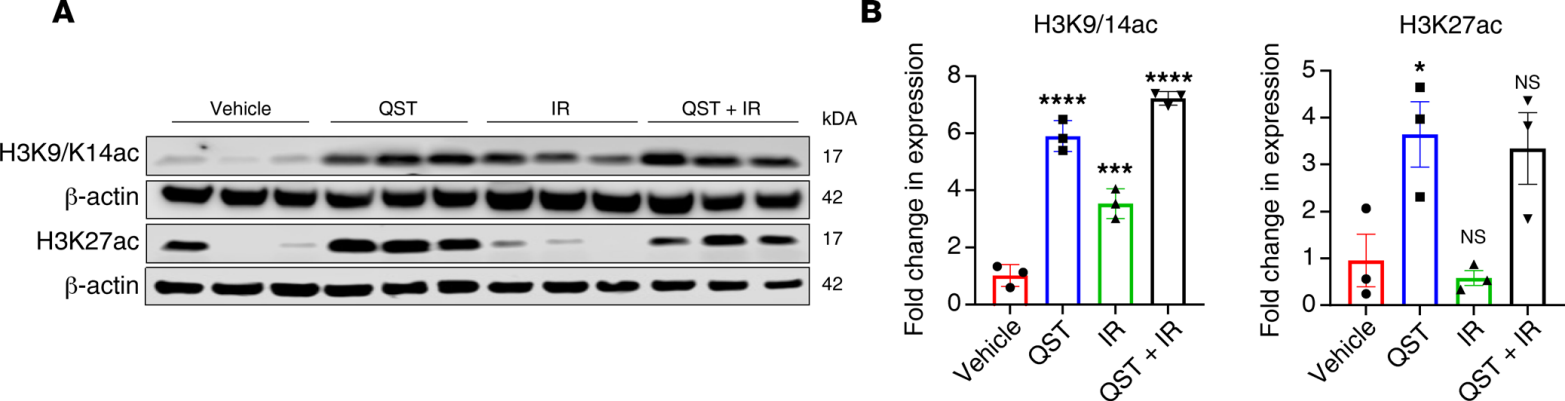
End-stage PD assessment of QST in an orthotopic PDX model of GBM. (**A**) Immunoblotting of protein lysates derived from homogenized brain tumors from each cohort (*n* = 3 mice per cohort). Membranes were probed for H3K9/14ac, H3K27ac, and β-actin. (**B**) Normalized levels of H3K9/14ac and H3K27ac protein in all cohorts. Circles and squares indicate values, bars indicate mean values, and error bars indicate SEM. *P < 0.05, ***P < 0.001, ****P < 0.0001 by unpaired, 2-tailed Student’s *t* test. NS, not significant.

All mice included in the survival study were processed for end-point PK and PD analyses once moribund. Plasma, tumor, and contralateral brain tissues were harvested 3 hours after dosing with 10 mg/kg QST, allowing for direct comparison of long-term treatment with the PK-PD data collected from acute (1-week) treatment with QST or combination therapy (Figure 8A). As shown in Figure 8, E and F, PK analyses demonstrated that unbound QST accumulated in the tumors (mean 71.4 nM) and peritumoral brain tissue (mean 3.4 nM) over time. There were no significant differences in total or unbound drug concentrations in tumor or brain tissue between the monotherapy or combination therapy cohorts (Figure 8, E and F). Immunoblot analysis of dissected tumor samples confirmed that QST induced substantial histone H3 hyperacetylation in brain tumors, compared with untreated animals, consistent with an in vivo on-target effect (Figure 9, A and B). Our results indicate that QST is a brain-penetrant drug that accumulates in both normal brain and tumor tissue.

### Induction of cell cycle arrest and a neuron-like cell fate with combined QST and IR treatment.

**Figure 10:**
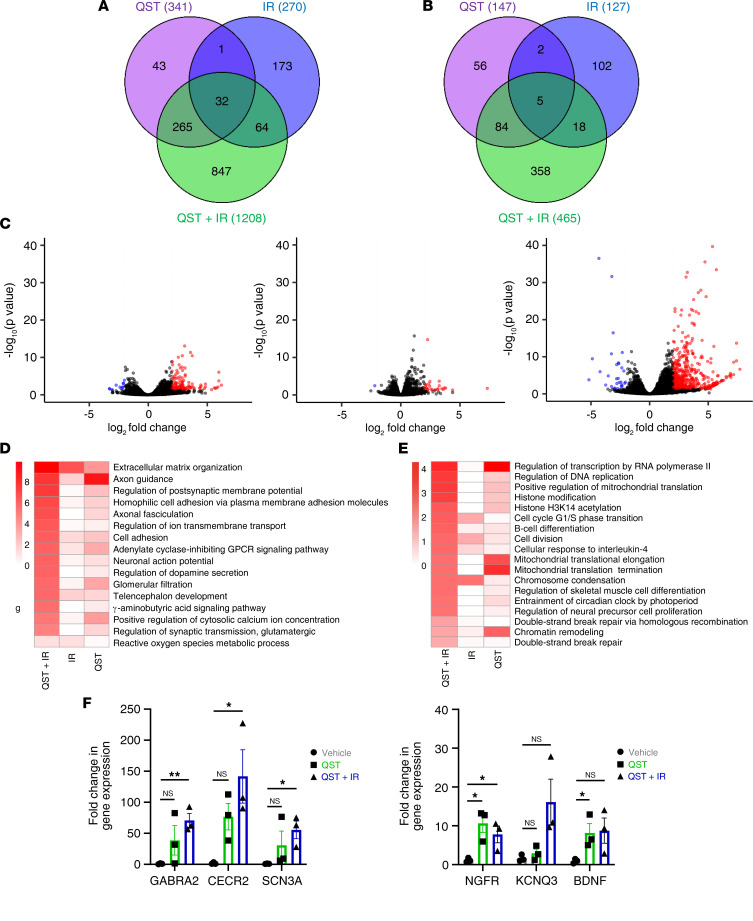
Combined treatment with QST and IR induces cell cycle arrest and a neuron-like cell fate in vivo. Venn diagrams show overlap in genes upregulated (**A**) or downregulated (**B**) in response to QST monotherapy, IR, or combination treatment (QST+IR). Gene numbers in each section are shown in parentheses. (**C**) Volcano plots showing the –log_10_(*P* value) and log_2_(fold change) for transcripts detected by RNA-seq analysis of endpoint orthotopic GB126 xenograft tumors treated with QST (left), IR (middle), or QST+IR (right). Significantly up- and downregulated genes (false discovery rate < 0.05, 2-fold) are marked in red and blue, respectively. GO analysis of genes upregulated (**D**) or downregulated (**E**) in GB126 tumors due to QST, IR, or QST+IR treatment. (**F**) Reverse transcription quantitative real-time PCR analysis of the expression of neuronal genes in GB126 tumors treated with either QST monotherapy or combination therapy. Circles, squares, and triangles indicate values; bars indicate mean values, and error bars indicate SEM. *P < 0.05, **P < 0.01 by unpaired, 2-tailed Student’s *t* test. NS, not significant.

To better understand the downstream effects of the various treatment regimens, we performed RNA sequencing (RNA-seq) to analyze the transcriptomes of GB126 tumors of mice treated with either acute (1-week) or prolonged QST, IR, or combination treatment. Acute QST (1-week) treatment resulted in significant transcriptional changes relative to vehicle controls (908 upregulated and 396 downregulated genes) (Supplemental Figure 6, A–C). Conversely, long-term QST led to modest changes in gene expression (341 upregulated and 147 downregulated genes) (Figure 10, A and B). Short-term fractionated IR (2 Gy administered in 3 doses MWF; Figure 7A) resulted in minimal changes in gene expression compared to vehicle treatment (Figure 10, A and B). Conversely, long-term combination treatment induced much more pronounced changes in gene expression, with 1208 upregulated and 465 downregulated genes (false discovery rate < 0.05, 2-fold; Figure 10, A and B). These data suggest that, although positive and negative changes in the expression of some genes are shared across all cohorts in end-stage tumors (Figure 10A), the combination-treated tumor transcriptomes are largely distinct from those of tumors that received QST or IR alone (Figure 10C).

To determine the biological processes that are differentially enriched in treatment groups, we performed gene ontology (GO) analysis on upregulated and downregulated data sets from each treatment group (35). In acutely treated tumors, GO analysis confirmed that combination treatment upregulated genes related to responses to cellular stress (oxidative stress, wound healing, and unfolded protein response), apoptosis, and cellular differentiation (Supplemental Figure 8D). QST resulted in similar responses, albeit not as pronounced (Supplemental Figure 6D). Furthermore, our analyses revealed that combination treatment and, to a smaller degree, QST monotherapy, resulted in significant downregulation of genes involved in the repair of DNA DSBs, cell division, and chromatin organization (Supplemental Figure 6E). Our results thereby establish that QST, especially when combined with IR, induces strong transcriptional changes in tumors that promote cellular differentiation, cell cycle arrest, and cell death (Supplemental Figure 6, A–D).

**Figure 11:**
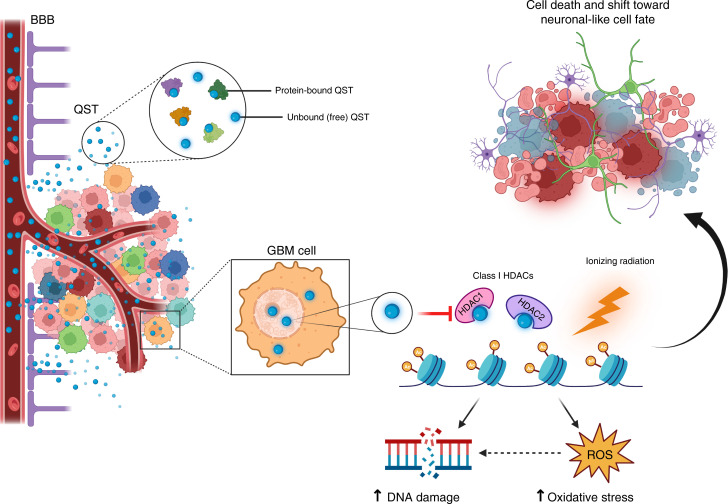
QST is a brain-penetrant HDACi that sensitizes GBM cells to radiation treatment. Summary of the major findings in this study. Through a careful PK-PD–guided approach, we determined that QST can cross the BBB and exert its intended PD effect (increase in histone acetylation) in normal brain tissue as well as GBM cells. A second-generation HDACi, QST has high subnanomolar isoform selectivity for HDAC1 and HDAC2, which are class I HDAC isoforms that are primarily responsible for mediating histone deacetylation. Free (non–protein bound) QST inhibit the function of HDAC1 and HDAC2, resulting in widespread histone hyperacetylation in GBM cells. QST treatment alone in GBM cells also results in increased levels of DNA damage and oxidative stress. When QST is combined with IR treatment, genes involved in DNA damage repair pathways and cell division are downregulated, whereas genes that regulate neuronal development and function are significantly upregulated. These findings suggest that combination therapy of QST and IR provides therapeutic benefit through decreased cell proliferation, dampening of pathways involved in the DNA damage repair response, and a shift toward a neuron-like cell fate. ROS, reactive oxygen species.

Interestingly, in end-stage tumors, we found that combination treatment induces striking upregulation of multiple genes associated with neuronal development, growth, function, and identity (e.g., axon guidance, axonal fasciculation, neuronal action potential, regulation of dopamine secretion, telencephalon development, γ-aminobutyric acid signaling pathway; Figure 10D). Similar changes were more modestly induced by QST but not by IR (Figure 10D). These results strongly suggest that QST treatment causes glioma cells to differentiate and adopt a neuron-like cell fate and that these transcriptional changes are amplified when the drug is combined with IR (Figure 10D). Moreover, GO analysis of downregulated genes found that the combination treatment group experienced substantial downregulation of genes associated with transcription, DNA replication, the cell cycle, chromatin remodeling, and DNA repair (Figure 10E). Expression of the top upregulated genes was validated by reverse transcription quantitative PCR, confirming that combination-treated and QST-treated tumors upregulated genes that are normally in expressed neurons (*GABRA2*, *CECR2*, *SCN3A*, *NGFR*, *KCNQ3*, and *BDNF*) (Figure 10F). We additionally confirmed increased expression of brain-derived neurotrophic factor (BDNF) precursor protein in the brain tumors of mice receiving combination therapy, compared with vehicle-treated controls (Supplemental Figure 6F). Taken together, these analyses suggest that combination treatment reduces cell proliferation and induces GBM cells to adopt a neuron-like cell fate (Figure 10, D and F, and Figure 11).

## Discussion

Systemic inhibition of HDACs is a branch of epigenetic therapy that has been investigated in clinical trials for GBM but has so far yielded disappointing results (2, 36, 37). Vorinostat, romidepsin, and panobinostat are the only pan-HDACis that have been tested clinically for GBM as either monotherapies or in combination with IR and/or temozolomide or bevacizumab. All 3 were ineffective and did not provide a survival benefit when compared to historical control data from previous phase II clinical trials (38–40). The clinical failure of HDACis for GBM treatment is attributable to their limited ability to cross the BBB, their high toxicity profiles, and their resultant narrow therapeutic windows (2, 41, 42). Notably, these HDACis advanced to clinical trials without PK and target-modulation analyses in the CNS of preclinical models of aggressive gliomas (43–46), all of which are valuable data necessary for effective clinical translation (43–45).

Several second-generation HDACis have been developed with higher isoform selectivity, with the aim of decreasing toxicity and increasing specificity, and they warrant preclinical investigation for the treatment of GBM (8). Individual HDAC enzymes harbor nonredundant, isoform-specific roles in different kinds of cancers, and it is hypothesized that HDACis with greater isoform selectivity may possess a higher therapeutic index and have fewer adverse effects (47). Because we recently discovered that HDAC1 promotes the tumorigenic properties and survival of GSCs, we questioned whether a brain-penetrant HDACi with higher affinity for HDAC1 would be effective in slowing tumor growth in vivo. Therefore, we investigated the translational potential of QST, an HDACi that is more selective against class I HDACs and exhibits marked potency toward HDAC1 and HDAC2, in preclinical models of GBM. We performed a comprehensive PK-PD correlation analysis for QST in preclinical in vitro and in vivo models of GBM. To our knowledge, this study is the first of its kind for any HDACi studied in the field of neuro-oncology.

We demonstrate that QST is highly cytotoxic to human GSCs and functions as a potent radiosensitizer in an orthotopic PDX model of GBM. Importantly, we performed brain- and tumor tissue–specific PK analyses of total and unbound QST concentrations and demonstrated that QST is a brain-penetrant molecule. Our findings are important given the role of HDACis in cancer therapy and the controversy surrounding QST’s efficacy for malignant brain tumor treatment. One study in a sonic hedgehog–driven medulloblastoma model reported that QST treatment had a statistically significant but modest survival benefit, whereas studies in a syngeneic model of GBM and a PDX model of DIPG reported no therapeutic benefit (14–16). It was previously suggested that QST’s failure to prolong survival in vivo could be attributed to its inability to cross the BBB, but these studies lacked data on direct drug levels and evidence of on-target engagement in the brain (14, 15). To this end, we used an elaborate PK-PD paired analysis approach to establish the brain-penetrant properties of QST and guide interpretation of the translational potential of this HDACi in GBM. We found that, when dosed at 10 mg/kg via IP injection, unbound levels of QST can be detected in normal brain tissue and that this concentration is sufficient to induce substantial histone H3 hyperacetylation. These results provide experimental evidence that QST can cross the BBB and induce its intended PD effect in the CNS, in contrast to previous conclusions based on survival benefit alone.

We show that, although QST slows the growth of intracranial GBM, monotherapy results in only a modest survival benefit. However, considering that numerous HDACis have been shown to display radiosensitizing properties in other malignancies (48–52), we questioned whether QST could enhance IR-induced cell death in GBM. We found that low nanomolar doses of QST robustly synergized with IR across multiple glioma cell lines. Importantly, we found that QST and IR combination therapy resulted in a significant extension of median survival compared with untreated and monotherapy regimens. To our knowledge, this is the first report demonstrating that QST can act as a potent radiosensitizer in vitro and in vivo in any preclinical cancer model. Hence, our results have important implications for the management of other malignancies, such as prostate, colon, lung, and esophageal cancer, wherein class I HDACs are frequently overexpressed and where IR is commonly used as a treatment modality (53, 54). Furthermore, our results strongly suggest that QST should be reevaluated as a potential radiosensitizer in preclinical models of DIPG, given that IR is currently the only treatment option available for children diagnosed with this aggressive and fatal glioma (55). Our results also strongly suggest that combinatorial treatment with QST and IR not only represses genes involved in cellular proliferation but also induces genes involved in neuronal signaling in vivo. In line with our observations, 2 recent studies in preclinical models of DIPG (with the bifunctional LSD1/HDAC inhibitor Corin) and GBM (with the small molecule MS-275) demonstrated that HDAC inhibition induces the expression of genes related to neuronal differentiation in vitro and in vivo (56, 57). Whether this shift toward neuron-like cell fate represents an induction of differentiation phenotype or a potential means to evade cell death remains an open question.

We found that QST treatment resulted in elevated levels of DNA DSBs, both in vitro and in vivo. It is well established that other hydroxamic acid-based HDACis (trichostatin A, vorinostat, panobinostat, and belinostat) can induce DNA damage and negatively regulate the DNA damage response pathway (53, 58–61). The precise mechanisms through which HDACis directly induce DNA damage and synergize with IR remain unclear, but several explanations have been proposed. Previous studies have shown that HDACi treatment can lead to the accumulation of reactive oxygen species, which can result in oxidized DNA base lesions (62–64). If left unrepaired, the oxidative stress–induced single-strand DNA breaks can be converted to DSBs during DNA replication (65, 66). Numerous studies have shown that HDACi treatment across various cancer cell lines also results in the transcriptional downregulation of genes involved in homologous recombination and nonhomologous end-joining (*Ku70*, *Ku86*, *DNA-PKcs*, *RAD51*, *BRCA1*, and *BRCA2*), which are critical for DSB repair (26, 53, 63). Our RNA-seq analysis of tumors receiving both QST and IR revealed that this combinatorial regimen resulted in the downregulation of genes involved in double-strand DNA repair, homologous recombination, and increased response to oxidative stress. These data suggest that HDACi-induced reactive oxygen species generation and dampening of the DNA damage response may contribute to DSB accumulation in GBM cells.

Another proposed mechanism for DNA damage is through histone hyperacetylation from HDACi treatment resulting in drastic structural changes in chromatin, exposing large portions of DNA to genotoxic agents (67, 68). It is hypothesized that combination treatment of HDACi and IR synergize by inducing excessive DNA damage and subsequent apoptosis (28, 69). Both HDAC1 and HDAC2 have also been shown to harbor important roles in the DNA damage response pathway by promoting DSB repair (29, 70). One seminal study demonstrated that HDAC1 and HDAC2 localize to DSB sites and induce local chromatin condensation through histone deacetylation, repressing transcription and preventing transcription from interfering with DNA repair processes (29). HDAC1 and HDAC2 depletion rendered cancer cells hypersensitive to IR and resulted in diminished DSB repair capacity, particularly by nonhomologous end-joining (29). Indeed, we found that QST treatment alone led to a gradual accumulation of DNA DSBs and that these effects were further exacerbated when combined with IR. Hence, we speculate that the radiosensitizing effects of QST in GSCs may be partly driven through potent inhibition of HDAC1 and HDAC2, against which QST exhibits the highest isoform selectivity (IC_50_: 0.1 nM and 0.3 nM, respectively) (8).

An important limitation inherent in our PDX models is the lack of an intact immune system, which could affect the response to treatment. Brain IR induces widespread and chronic neuroinflammation, which can compromise BBB integrity, cognition, and cell survival (71–73). The radiosensitizing effects of QST will need to be studied, either in syngeneic or transgenic GBM models. Moreover, future studies should address whether using lower doses or different dosing regimens of IR may enhance the synergism with QST in vivo.

To date, no hydroxamic acid–based HDACis have been shown to harbor radiosensitizing properties in preclinical models of GBM. However, it remains unclear whether these inhibitors failed to provide any therapeutic benefit because of inadequate brain penetration or insufficient on-target modulation. Our study therefore emphasizes the importance of implementing a PK-PD–guided approach when evaluating or developing new drugs for GBM. Although PK analyses are now commonly performed in preclinical trials for a variety of different brain tumors, these studies typically measure only the total brain-to-plasma concentration ratio as a measure of drug-brain penetration (74–76). However, the value of this ratio is limited and may lead to erroneous conclusions because it does not consider the protein- or lipid-unbound fraction of drug in the plasma and the brain (77). Therefore, we employed an equilibrium dialysis method combined with LC-MS/MS analysis to measure the unbound brain-to-plasma concentration, which represents the pharmacologically active fraction of a drug. A crucial finding in our study was the species-specific differences in QST stability. QST is highly unstable in mouse plasma and brain but highly stable in both human plasma and brain. A recent study reported that hydroxamic acids such as QST are common substrates of a family of esterases (carboxylesterases) that are abundantly present in rodent plasma but absent in human plasma (34, 78). We confirmed that the addition of BNPP, a specific inhibitor of carboxylesterases, stabilized QST in mouse plasma. Our results therefore highlight a large discrepancy in the metabolic stability between rodent and human species. This is an important consideration for translating preclinical studies to the clinic because such differences may hinder further clinical development of promising drug candidates. Our data will thereby serve as a valuable resource and note of caution for future preclinical studies employing QST or similar drugs with species-specific stability.

QST has been tested in phase I and II clinical trials for lung, ovarian, and breast cancer, and it was well tolerated at a maximum-tolerated dose of 12 mg given 3 times weekly (79). Notably, QST harbors superior clinical tolerability compared with panobinostat, another HDACi currently in trials for aggressive gliomas. Data from phase I trials found that, although hematologic toxicities (grade 1 and 2) were rare in patients treated with QST (<5%), such toxicities were far more common and severe (grade 3 and 4) during treatment with panobinostat (5, 6, 79, 80). Although the tolerability of QST in combination with other agents in humans remains unexplored, our preclinical data suggest that combinatorial treatment with fractionated IR is well tolerated. Because QST is an HDACi that has passed phase I clinical trials in several cancers and is well tolerated in humans, it is a promising candidate for phase 0 “trigger” trial testing in patients with GBM (79). This approach would enable characterization of the PK-PD relationship of QST in humans and fast-track development of QST as an adjuvant therapy for patients with GBM.

Identification of drugs that can enhance the effects of radiation treatment is an intense area of research within neuro-oncology, especially for patients with *MGMT*-unmethylated tumors. Nevertheless, although the use of radiosensitizers represents a promising strategy in GBM, the development of these novel agents has been underwhelming (81). Here, we provide the first preclinical report to our knowledge for a brain-penetrant HDACi, QST, with potent radiosensitizing properties. Future investigation is required to elucidate the molecular consequences of QST treatment and its synergistic relationship with radiation-induced DNA damage in GSCs. Overall, our results provide a rationale for developing QST as a potential combination therapy with IR for GBM treatment.

## Methods

### Primary cell culture.

Patient-derived GSC lines (GB187, GB239, GB282, GB71, GB82, and GB126) were established from resected primary GBM tumor tissue at the Barrow Neurological Institute. Cell lines generated at the Barrow Neurological Institute were genetically profiled for mutations and copy number aberrations using the IvySeq custom gene panel established at the pharmacodynamics core at the Ivy Brain Tumor Center in Phoenix. BT145 GSCs were obtained from Keith Ligon’s laboratory at the Dana-Farber Cancer Institute (Boston, Massachusetts, USA). All human GSCs were cultured as described elsewhere (11). U87-MG cells (HTB-14) were purchased from the American Type Culture Collection and grown according to the manufacturer’s recommendations. GSCs were cultured as spheres on non–tissue culture–treated 10-cm plates or adherent cultures on laminin on tissue culture–treated 10-cm plates (Thermo Fisher Scientific). GSCs were grown in DMEM/nutrient mixture F-12 media, supplemented with B27, N2 (Invitrogen, Thermo Fisher Scientific), and 1% penicillin-streptomycin in the presence of 20 ng/mL epidermal growth factor and basic fibroblast growth factor (MilliporeSigma).

### Cell viability assays after QST treatment.

GSCs were seeded in laminin-coated tissue culture–treated 96-well plates (clear bottom, white plate; Corning) at a density of 1000–5000 cells per well (cell line dependent) in GSC media. U87-MG were seeded using their normal growth conditions without laminin (10% bovine calf serum in DMEM). All cells were incubated at 37°C and allowed to adhere overnight. The next day, cells were treated with incremental concentrations of QST (Selleckchem; 0, 10, 25, 50, 100, 250, 500, and 1000 nM) diluted in media. Cells treated without QST were treated with DMSO diluted in media. Following treatment with QST, cells were grown for 3 to 5 days (cell line dependent), and cell viability was measured and quantified. All cell viability measurements were performed using the CellTiter-Glo luminescent cell viability assay (Promega) following the manufacturer’s instructions. For the sulforhodamine B colorimetric assay for cytotoxicity, the protocol was performed as described elsewhere (33). All cell viability results represent the mean of at least 3 biological replicates, each containing 3 technical replicates.

### Orthotopic xenograft studies.

Seven-week-old *Foxn1^nu^* nude male mice (The Jackson Laboratory) were used for in vivo orthotopic transplantation of luciferized GB126 (male) cells (at a density of 50,000 cells/μL). All procedures were performed as described elsewhere (11). All mice were observed daily and were sacrificed upon the onset of severe neurological symptoms and more than 10% body weight loss. Survival data were plotted and analyzed using GraphPad Prism 8. See Supplemental Methods for detailed in vivo methods.

### Bioanalytical LC-MS/MS method.

QST concentrations in specimens were measured using reverse-phase LC on the AB SCIEX QTRAP6500+ LC-MS/MS system by operating electrospray in the positive ion mode. For liquid chromatographic separation, gradient elution was performed using a Phenomenex Kinetex F5 100 Å column (100 × 2.1 mm, 2.6 μm). The initial composition of the mobile phase was 70% phase A (0.1% formic acid in water) and 30% phase B (0.1% formic acid in 1:1 acetonitrile/methanol) with a 0.35 mL/min flow rate. Gradient elution was achieved as follows: mobile phase (B) was maintained at 30% from 0 to 0.3 minutes, increased to 95% from 0.3 to 0.8 minutes, maintained at 95% from 0.8 to 2.5 minutes, and decreased to 30% from 2.5 to 2.8 minutes. The total run time was 3.5 minutes. The internal standard used in this study was D_8_-infigratinib. The retention times for QST and D_8_-infigratinib were 1.6 and 1.8 minutes, respectively. Mass-to-charge ratio transitions were as follows: 395.20 → 144.00 (QST) and 568.08 → 321.00 (D_8_-infigratinib). LC-MS/MS analysis was performed using an Analyst 1.7 Chromatographic Data System. See Supplemental Methods for detailed PK experiments.

### RNA-seq analysis.

To analyze RNA-seq data, raw RNA-seq reads were selected for quality and length by removing low-quality reads and adapter sequences using cutadapt. Samples were then aligned to 2 separate genomes, GRCm39 (mouse) and GRCh38.p13 (10), using STAR (82). Samples were filtered with XenofilteR using human BAM as graft and mouse as host to reduce mouse DNA in samples compared. Count tables were generated using featureCounts in the Subread package (83), and the resulting count tables were analyzed in R using DESeq2 (https://github.com/thelovelab/DESeq2) to identify differentially expressed genes. Genes that were up- or downregulated at least 2-fold with a false discovery rate less than 0.05 were considered differentially expressed for downstream analyses. After identifying differentially expressed genes, GO analyses (35) were performed using the Fisher method (cutoff, *P* < 0.01) to identify gene categories that were up- and downregulated by the various treatments. Study data have been deposited in the NCBI Gene Expression Omnibus (GEO) (84) and are available through accession number GSE241227 (https://www.ncbi.nlm.nih.gov/geo/query/acc.cgi?acc=GSE241227).

### Statistics.

Data are presented as the mean and SEM. If comparing 2 conditions or cell lines, significance was tested with unpaired, 2-tailed Student’s *t* test. Significance of differences between conditions or cell lines was tested with 2-way analysis of variance (ANOVA) with Bonferroni’s multiple-comparison test using GraphPad Prism 9. Survival studies were analyzed using the Kaplan-Meier method with the Mantel-Cox log-rank test (Prism 9). Statistical significance was defined as *P* less than 0.05.

### Study approval.

The patient samples used for this research were provided by the Biobank Core Facility at St. Joseph’s Hospital and Medical Center and Barrow Neurological Institute. The samples were deidentified and conformed to the Biobank Institutional Review Board’s protocol. Animal husbandry was performed in accordance with the guidelines of the St. Joseph’s Hospital and Medical Center and Barrow Neurological Institute under the protocol approved by the Institutional Animal Care and Use Committee.

### Data availability.

All data are available within the article and can be accessed from public repositories or in the Supporting Data Values file.

## Author contributions

CLC, AT, and SM conceived and designed all experiments. CLC, ELM, JBM, and CIW standardized the techniques, performed in vitro and in vivo experiments, and analyzed the data. TM, WK, and AT performed all analyses and developed methods for pharmacokinetic studies. SG performed in vitro experiments and assisted with data analysis. ZO assisted with in vivo experiments. CC provided guidance and support for FACS analysis. WY performed and helped with statistical analyses. TM, ELM, JBM, CIW, ZO, NS, and AT edited the manuscript. NS provided patient tissues to establish patient-derived glioma cell lines. SM and AT coordinated and supervised the project. CLC and SM wrote the manuscript.

## Supplementary Material

Supplemental data

Supporting data values

## Version 1 11/22/2023

Electronic publication
