# Supplementary material for: Quisinostat is a brain-penetrant radiosensitizer in glioblastoma
Source: JCI Insight. 2023 Nov 22;8(22):e167081. doi: 10.1172/jci.insight.167081 (PMC10721329; doi:10.1172/jci.insight.167081)
Supplement: Supplemental data [file jciinsight-8-167081-s140.pdf]

SUPPLEMENTAL MATERIALS

SUPPLEMENTAL METHODS

Western Blotting

Cellular protein from cultured cells was homogenized in radioimmunoprecipitation assay (RIPA) lysis buffer containing protease and phosphatase inhibitors (ThermoFisher Scientific), rotated at 4 °C for 20 minutes and then centrifuged at 15,000 rpm for 10 minutes at 4 °C. For histone extractions, benzonase (0.1 U/µL) was added in RIPA buffer (Millipore-Sigma, 70746-4). Protein concentrations from whole-cell extracts were determined using the Bradford Protein Assay (ThermoFisher Scientific). Equal amounts of protein (10-40 µg/lane) were loaded onto 10% or 12.5% sodium dodecyl sulfate–polyacrylamide gel electrophoresis gels and transferred to a polyvinylidene fluoride membrane (Millipore-Sigma).

Cellular protein from frozen tissue of non–tumor-bearing mouse brains, tumor-bearing mouse brains, and flank tumors were homogenized in a prechilled glass tissue grinder (VWR) with RIPA lysis buffer containing protease and phosphatase inhibitors (ThermoFisher Scientific), with 500 uL of RIPA buffer for 10 mg of tissue. Once homogenized, the tissue lysates were kept on ice for 30 minutes and vortexed every 10 minutes. The samples were then centrifuged at 15,000 rpm for 10 minutes at 4 °C to collect the protein lysates.

Membranes were blocked with 5% nonfat milk for 1 hour at room temperature and incubated overnight with primary antibody at 4 °C. Primary antibodies used were rabbit anti-p21 (1:500; Abcam, ab109520), rabbit anti-gamma H2AX (phospho Ser139; 1:1000, Abcam, ab11174), rabbit anti-H3K27ac (2 µg/mL, Abcam, ab4729), rabbit anti-H3K9/14ac (1:1000, Cell Signaling Technologies, 9677), mouse anti-Histone H3 (1:1000, Cell Signaling 14269), mouse

anti-GAPDH (1:1000, Cell Signaling Technologies, 97166), rabbit anti-cleaved poly (ADP-ribose) polymerase Asp 214 (1:1000, Cell Signaling, 9541), rabbit anti-BDNF (1:1000, Abcam, ab108319) and mouse anti- β-actin (1:1000, Bio-Rad, MCA5775GA). Membranes were probed with fluorophore-conjugated anti-mouse or anti-rabbit secondary antibodies (1:10,000; ThermoFisher Scientific). Western blots were developed using the LI-COR Odyssey CLx imaging system (LI-COR) and quantitated using the Image Studio Lite software. All Western blots are representative images from a minimum of 3 biological replicates.

Immunocytochemistry

Cells were grown as adherent cultures on laminin-coated glass coverslips (Thermo Fisher Scientific) in GSC media. Twenty-four hours after plating, the cells were treated with QST or DMSO diluted in GSC media. Seventy-two hours after treatment, cells were fixed with 4% paraformaldehyde for 13 minutes at room temperature. Cells were washed with phosphate-buffered saline (PBS) and subsequently permeabilized and blocked with 5% normal goat serum (Sigma Aldrich) and 0.2% Triton X-100 in PBS (blocking solution) for 30 minutes at room temperature. The cells were incubated with primary antibodies overnight at 4 °C in blocking solution. Primary antibodies used in this study included rabbit anti-Ki67 (1:1000; Abcam, 15580), rabbit anti-Cleaved Caspase 3 (1:400; Cell Signaling Technologies, 9661), rabbit anti-gamma H2AX (phospho Ser139; 1:1000, Abcam, ab11174), and mouse anti-human Nestin (1:500; Novus Biologicals, 10C2). The following day, the cells were washed with PBS 3 times, incubated with fluorophore-conjugated secondary antibodies at 1:1000 dilutions (Alexa Fluor 568 goat anti-mouse, Abcam, ab175473; Alexa Fluor 488 goat anti-rabbit, Abcam, ab150077) for 1 hour at room temperature, and washed in PBS 3 more times. Cells were mounted onto

SuperFrost Plus microscope slides using Fluoroshield Mounting Medium containing DAPI (Abcam). Images were acquired using a confocal microscope (Leica Microsystems; TCS SP5) operated with Leica Application Suite software. The fraction of Ki67- and Cleaved Caspase 3–positive cells were counted from 5 independent images from each condition. The mean and standard deviation were calculated from 3 biological replicates for all control and QST-treated experiments.

Image Acquisition

Analysis of immunostaining of cultured GSCs was performed on confocal stacks (with a step size of 0.5–1.5 μm) acquired with either a 20× water-immersion objective or a 63× oil-immersion objective on a laser-scanning confocal microscope (Leica Microsystems; TCS SP5) operated with Leica Application Suite software. All images were processed using ImageJ software (National Institutes of Health).

Flow Cytometry

For cell cycle analysis following short-term DMSO or QST treatment in GSC cultures, approximately 1 million cells were harvested, washed in cold 1× PBS once, and fixed with 3–4 mL of ice cold 70% ethanol, added dropwise while vortexing. Cells were fixed on ice for 30 minutes and washed twice with 1× PBS. Cells were then resuspended in 500 μL of propidium iodide/RNAse staining buffer (BD Biosciences, 550825) for 15 minutes at room temperature before analysis. Data from all the samples were obtained using a Fortessa flow cytometer (BD Biosciences) and analyzed using the FlowJo software (TreeStar). Cell cycle analysis was performed in triplicate for all cell lines.

For apoptosis analysis, the Dead Cell Apoptosis Kit with Annexin V FITC and Propidium Iodide for Flow Cytometry (Thermo Fisher Scientific, V13242) was followed using the manufacturer instructions, with the exception that we employed ViaDye Red Fixable Viability Dye Kit (Cytek, SKU R7-60008) instead of propidium iodide to label necrotic cells. Samples were run on the AURORA 3L 24 color spectrum cytometers (Cytek), unmixed by SpectroFlo application, and analyzed with FlowJo 10.8.1 software (Beckton Dickinson). Recommended negative controls for flow cytometry include (1) no Annexin V FITC and no ViaDye Red, (2) Annexin V FITC alone, and (3) ViaDye Red alone. The following gating strategy was used: debris population was excluded as the subset of cells with low forward scatter in the forward scatter area versus side scatter area plot. After excluding the debris, cells were gated on the Annexin versus ViaDye Red log plot with the classification as live (Annexin V negative, ViaDye Red negative), early apoptotic (Annexin V positive, ViaDye Red negative), late apoptotic (Annexin V positive, ViaDye Red positive), or necrotic (Annexin V negative, ViaDye Red positive). The frequency of the different cell subsets was reported as frequency on the total cell population, excluding debris.

In Vitro Irradiation Studies

For all in vitro radiosensitization experiments involving treatment with IR using the RS 2000 irradiator (Rad Source) or the X-Rad225XL irradiator (Precision X-Ray), GSCs were plated on laminin-coated tissue culture–treated 96-well plates and incubated at 37 °C overnight for 24 hours. The next day, cells were pretreated with QST or an equivalent volume of DMSO for 1 hour and then irradiated with various doses of IR (cell-line dependent). Cell viability was measured as described above using the CellTiter-Glo assay (Promega) 3 to 5 days after

treatment. For experiments involving protein characterization of IR-treated cells preceded by treatment with QST, whole-cell lysates were collected at 1, 2, 6, and 24 hours after IR. Radiation was delivered using a RS2000 series biological irradiator (Rad Source Technologies) or the X-Rad225XL irradiator (Precision X-Ray).

Flank Tumor Implantation

For flank implantations, cells were prepared in a 1:1 ratio with 100 uL Matrigel (Corning #356234) and 100 uL of a single-cell suspension of U87 in PBS (500,000 cells) in a 1 mL syringe fitted with a 26-gauge needle. Mice were anesthetized with isoflurane in a plastic desiccator placed in an externally vented fume hood. The U87-Matrigel cell suspension was then subcutaneously injected into the flank of the mouse on the posterior/lateral aspect of the lower rib cage. Mice were monitored daily, and growth of flank tumor area was measured with a digital caliper (ThermoFisher) twice a week. Mice were sacrificed once the tumor size grew to more than 2000 mm^3 in size.

Live Bioluminescence Imaging

Two weeks after implantation, mice were examined for tumor growth by monitoring bioluminescence every 7 days using the IVIS Xenogen Spectrum platform. D-Luciferin Potassium Salt (Gold Biotechnology) was dissolved in PBS at a final concentration of 15 mg/mL. All mice were weighed each week and administered D-Luciferin via an intraperitoneal injection (10 μl/g). Fifteen minutes after injection, the mice were sedated using gaseous isoflurane (Piramal) and placed inside an IVIS Spectrum in vivo imaging system (Perkin Elmer)

for bioluminescence imaging. The total flux (photons/second) within the region of interest was calculated using the Living Image software 4.5 (Perkin Elmer).

Preparation of QST for In Vivo Use

For in vivo preparation, QST was dissolved in 50% polyethylene glycol (PEG) 300, 50% sterile water solution for 10 mg/kg dosing. The suspension was then sonicated for 10 minutes to allow the drug to completely dissolve. Finally, the pH of both the drug and the vehicle solutions was adjusted to 7.4 before intraperitoneal dosing.

Determination of Optimal Administration Route

Foxn1^{nu} nude male mice (The Jackson Laboratory) were used to determine the drug administration route that would result in the best QST bioavailability. Three cohorts of mice were treated with a single dose of 10 mg/kg QST delivered through intraperitoneal, subcutaneous, or oral gavage routes (3 mice per cohort). Following administration of the single dose, approximately 30 µL of blood was drawn from the tip of the tails at the following time points: 0.5, 1, 2, 4, 6, 8, and 24 hours. The collected blood was immediately centrifuged at 3000 rpm for 10 minutes at 4 °C to separate plasma, which was subsequently flash frozen. At the 24-hour time point, following the last blood sample collection, mice were sacrificed and the whole brains from each mouse were dissected and flash-frozen for subsequent analysis.

In Vivo Irradiation Studies

Intracranial or flank tumor–bearing nude mice were sedated with gaseous isoflurane before IR. On the first week of treatment, 2 hours after treatment with QST or vehicle, the mice were

treated with 2 Gy of ionizing radiation on MWF for a total 6 Gy (3 doses). Ionizing radiation was administered with the RS2000 series biological research irradiator (Rad Source Technologies).

Treatment of Flank-Implanted Mice with QST and/or Radiation

Implanted flank tumors were allowed to grow until the tumor size reached 100 mm^3 volume. Mice were randomized into groups before treatment and underwent treatment on MWF for the entire duration of the experiment until the tumor volumes exceeded 2000 mm^3. Treatment groups included vehicle (50% PEG-300), QST alone (10 mg/kg), flank IR treatment with vehicle (2 Gy), and flank IR treatment (2 Gy) with QST (10 mg/kg). For mice receiving IR treatment with or without QST, mice were treated with 2 Gy on MWF for a total 6 Gy during the first week of treatment. QST was administered to mice through intraperitoneal injections 2 hours before flank tumor radiation treatment. Upon completion of the radiation regimen, IR-treated mice subsequently received QST or vehicle alone for the rest of the experiment. Tumor growth and treatment response were monitored by manually measuring the tumor area twice per week starting at 13 days after implantation. Upon reaching the tumor volume threshold (2000 mm^3), mice were sacrificed, and plasma and tissue samples were harvested for PD and PK analyses 2 hours after treatment with a final dose of QST (10 mg/kg).

Treatment of Mice with Intracranially Implanted QST and/or Radiation

Mice with implanted tumors were allowed to grow until the tumor bioluminescence score reached 10^8 radiance ($p/s/cm^3/sr$). Mice were randomized into groups before treatment. For survival studies, mice underwent treatment on MWF for the entire duration of the experiment until moribund. For mice receiving IR treatment with or without QST, mice were treated with 2

Gy on MWF for a total of 6 Gy in the first week of treatment. Treatment groups included vehicle (50% PEG-3000), QST (10 mg/kg) alone, 6 Gy whole-brain IR treatment with vehicle, and 6 Gy whole-brain IR treatment with 10 mg/kg QST. QST was administered to mice through intraperitoneal injections two hours prior to whole-brain radiation treatment. Upon completion of the radiation regimen, IR-treated mice subsequently received QST or vehicle alone for the rest of the experiment. Tumor growth and treatment response was monitored by IVIS bioluminescence once per week. For survival studies, mice were sacrificed, and samples were collected for PD and PK analyses once moribund 2 hours after treatment with a final dose of QST (10 mg/kg).

For short-term PK-PD correlation studies, tumor-bearing mice were randomized into groups and underwent a single week of treatment with QST (10 mg/kg) on MWF. For mice receiving IR treatment with or without QST, mice were treated with 2 Gy on MWF for a total of 6 Gy. QST was administered to mice through intraperitoneal injections 2 hours before whole-brain radiation treatment. On the third and last day of treatment, mice were sacrificed and processed for PD and PK analyses 3 hours after administration of QST or vehicle. For PD analyses, the mice were euthanized with isoflurane, and the tumors were dissected out of the brain and flash-frozen for subsequent analysis through Western blotting and RNA sequencing. Tissue from the hemisphere contralateral to the tumor was also collected as a normal brain/nontumor reference sample. For PK analyses, the mice were anesthetized with isoflurane, and at least 300 μL of blood was drawn from the right ventricle of the heart. The blood was collected in tubes containing 10 μL of 0.1 M KOH in EDTA to prevent blood coagulation. Blood samples were immediately centrifuged at 3000 rpm for 10 minutes at 4 °C to allow separation of plasma. After collection of the blood, the tumor was dissected out of the brain and flash-frozen. Both plasma and erythrocytes were flash-frozen for subsequent analysis.

Calibration Standards and Quality Control Samples

Stock solutions of standard (1 mM QST) and IS (250 µM D_8-infigratinib) were prepared in acetonitrile. Working solutions for calibration curve standards and quality controls (QCs) were prepared by dilutions with a 40% methanol mixture aqueous solution. The IS precipitation solution (10 nM) was prepared from the IS stock solutions by dilution with methanol. Calibration standards and batch qualifying QCs were freshly spiked for every batch. For sample analysis in human and mouse matrices, calibration standards were prepared in bulk by spiking appropriate amounts of working solutions into blank human plasma, used as a surrogate matrix due to the instability of QST in mouse plasma. For sample analysis in neural stem cell media and cell lysate, calibration standards were prepared in bulk by spiking appropriate amounts of working solutions into neural stem cell media. QC samples were prepared in bulk by spiking appropriate amounts of working solutions into blank mouse plasma or cell media. Preparation of calibration standards and QC samples was performed at 4 °C. Final concentrations range of the calibration standards were 1–1000 nM in human plasma or neural stem cell media. Three QC levels (low, medium, and high) were used during all sample analyses. The concentrations of QC samples in various matrices were 3 nM (low QC), 22 nM (medium QC), and 800 nM (high QC). All stock solutions and working solutions were stored at 4 °C.

Plasma Sample Preparation

Frozen plasma samples were thawed at 4 °C. An aliquot of 30 µL mouse plasma was transferred into a micro centrifuge tube, followed by 30 µL of blank human plasma, and precipitation with 180 µL of IS-containing methanol precipitation solution. The mixture was

vortex-mixed for 10 s and centrifuged at 12000 g at 4 °C for 10 min. A 100-μL aliquot of the supernatant was transferred to an autosampler vial, and 5 μL was injected into the LC-MS/MS system for analysis. Whenever necessary, appropriate dilutions were made to accommodate the analysis of small volume samples.

Brain and Brain Tumor Sample Preparation

Normal brain and brain tumor tissue homogenates were prepared by 1:4 (mass/volume) ratio with PBS. Samples were homogenized under 6.00 m/s speed for 40 seconds with 3 cycles by Bead Ruptor Elite homogenizer (Omni International). Plasma was used as a surrogate matrix for brain/tumor homogenate. Brain homogenate samples from in vivo studies were prepared as described for plasma samples. Analyte and IS were extracted by protein precipitation with methanol containing IS. After centrifugation at 12000 rpm for 10 minutes at 4 °C, 5 μL of supernatant was injected into the LC-MS/MS system for analysis.

Cell Media and Lysate Sample Preparation

Cell media and lysates were thawed at room temperature. An aliquot of 20 μL of cell media or lysate was transferred into a micro centrifuge tube followed by protein precipitation with 60 μL of IS-containing methanol precipitation solution. The mixture was vortex-mixed for 10 s and centrifuged at 12000 g at 4 °C for 10 min. A 50-μL aliquot of the supernatant was transferred to an autosampler vial, and 5 μL was injected into the LC-MS/MS system for analysis.

Stability Study in Mouse Plasma, Mouse Brain, Human Plasma, Human Brain, and Neural Stem Cell Media

The stability of QST was determined in BALB/c mouse plasma, male nude athymic perfused and nonperfused mouse brain homogenate (1:9 w/v of PBS; pH 7.4), human brain (obtained from VRL Eurofins) homogenate (1:4 w/v of PBS; pH 7.4), pooled human plasma (Innovative Research), and neurobasal media. QST stock solutions (1 mM) were prepared in acetonitrile, subsequently diluted in a 40% methanol mixture, and added to the matrices to make final concentrations of 100 nM or 10 nM. Then 50 or 30 µL of either plasma or brain homogenate containing QST were aliquoted into 1.5-mL microcentrifuge tubes (Eppendorf) and were incubated at either 4 °C or 37 °C for 0, 2, 4, 6, 12, or 24 hours (n = 3 at each time point). The stability of QST in mouse plasma was also tested at 0, 2, 8, 16, or 24 hours (n = 3 at each time point) in the presence of esterase inhibitors: 0.25 mM BNPP, 1mM dithiobis(2-nitrobenzoic acid) (DTNB), or 1.25 mM phenylmethylsulfonyl fluoride (PMSF). Plasma and brain homogenate samples were stored at −80 °C until LC-MS/MS analysis.

Reverse Transcription Quantitative Polymerase Chain Reaction

Total RNA was extracted from orthotopic tumors by using the RNeasy Mini Kit (Qiagen) in accordance with the manufacturer's instructions. RNA was quantified on a NanoDrop Spectrophotometer (Tecan), and 1 µg of total RNA was used for complementary DNA synthesis by using the SuperScript VILO kit (Life Technologies). Quantitative polymerase chain reaction was performed using inventoried TaqMan assays for respective target genes and housekeeping control genes (18S) on the QuantStudio 6 Flex Real-Time PCR System (Life Technologies). Fold change in gene expression was analyzed using the delta delta Ct method.

SUPPLEMENTAL FIGURES

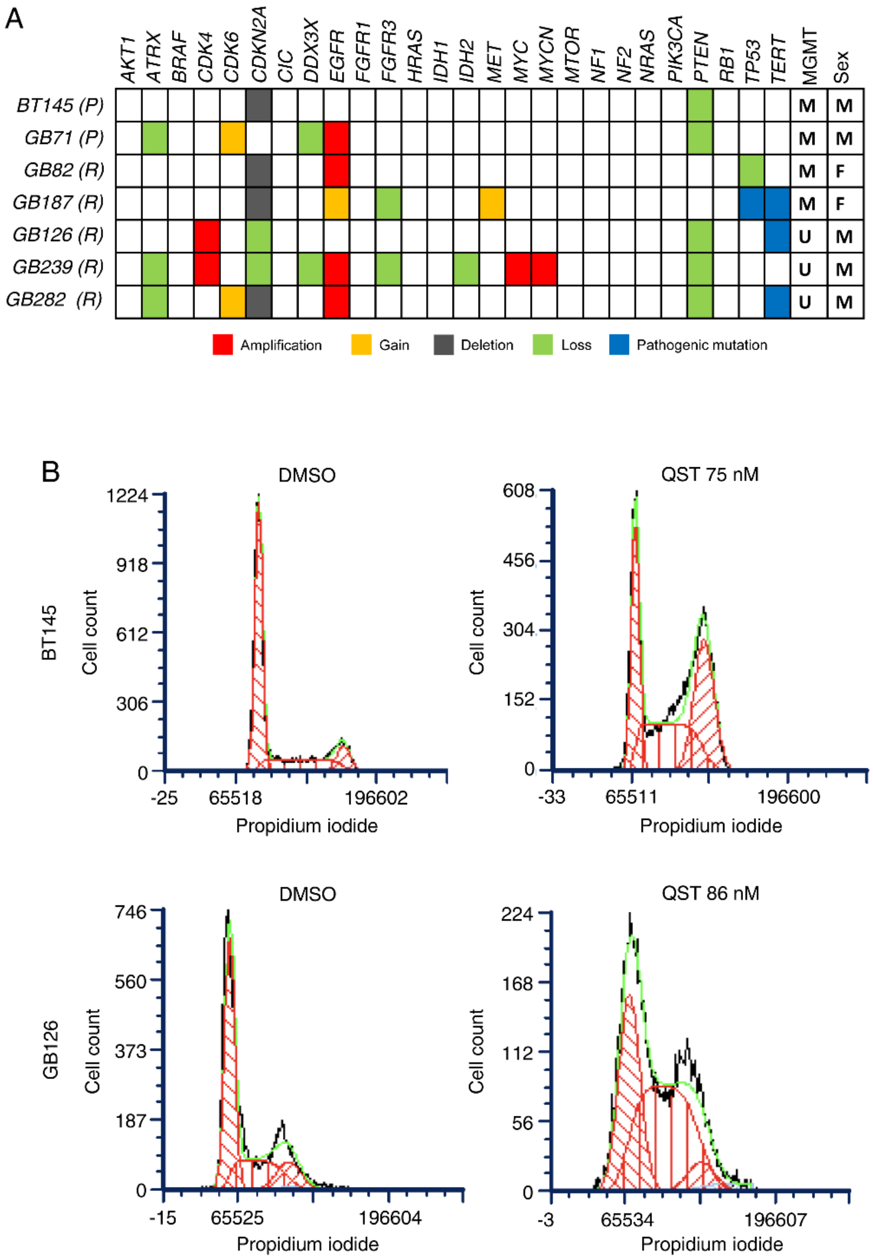

Supplemental Figure 1. Genomic characterization of cell lines used in this study and cell cycle analysis of QST-treated GSCs. **(A)** Patient-derived GSCs were sequenced and profiled for genetic mutation and copy number variation aberrations using the IvySeq custom gene panel developed at the Ivy Brain Tumor Center. P = primary glioblastoma; R = recurrent glioblastoma. Under *MGMT*, M = methylated and U = unmethylated. Under Sex, F = female and M = male. **(B)** Representative histogram plots depicting the proportion of cells in each phase of the cell cycle based on propidium iodide staining in BT145 and GB126 cells treated with DMSO or IC_{50} of QST for 24 hours. For each cell line, the data are compiled from at least 3 independent experiments.

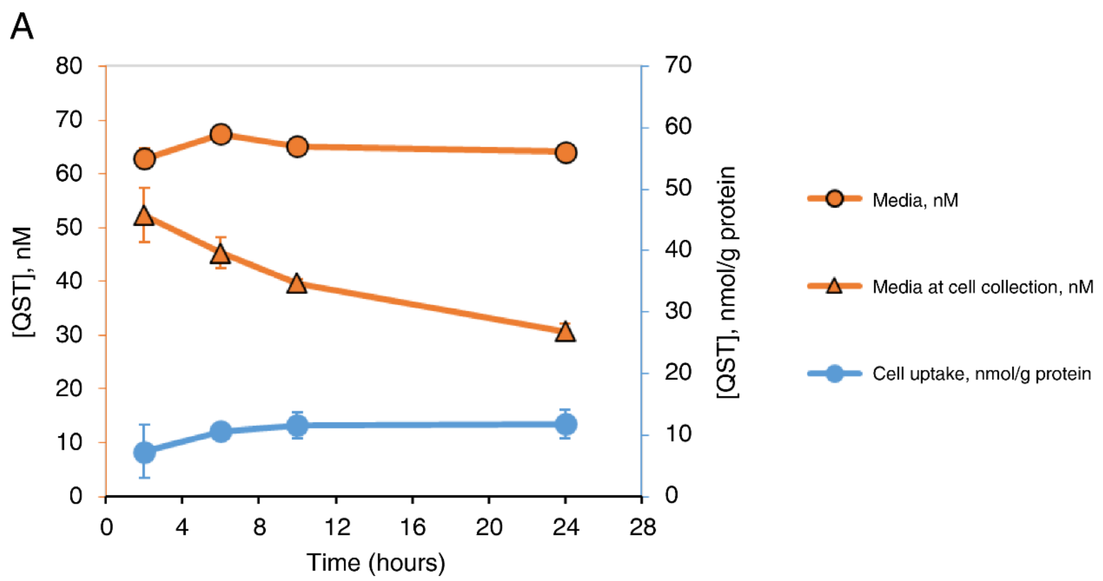

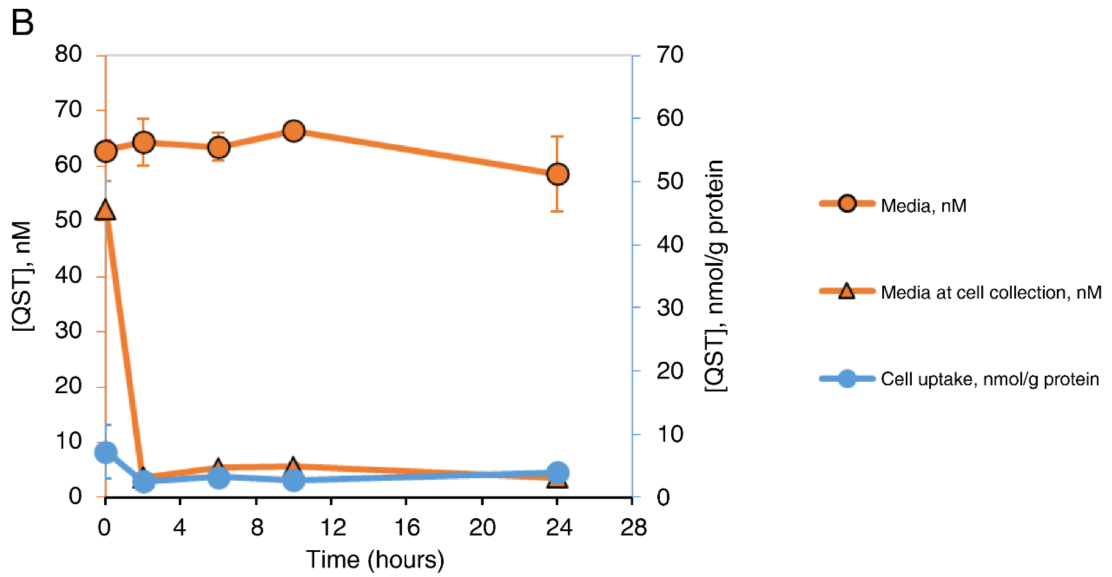

Supplemental Figure 2. Temporal dynamics of intracellular uptake of QST in BT145. **(A)** Levels of intracellular QST with continuous exposure to drug (75 nM) in BT145 over the course of 24 hours. Blue line indicates measured intracellular levels of QST at 2, 6, 10, and 24 hours after treatment. Orange line with triangles indicates levels of QST present in the cell media at each collected time point. Orange line with circles denotes the baseline levels of drug present when it was spiked into the cell media for each time point (*t* = 0 hr). **(B)** Levels of intracellular QST in BT145 cells that were treated with 75 nM QST and underwent drug washout 2 hours after initial treatment. Blue line indicates mean measured intracellular levels of QST at 2, 6, 10, and 24 hours after drug washout. Orange line with triangles indicates mean levels of QST present in the cell media at each collected time point. Orange line with circles denotes the mean baseline levels of drug present when it was spiked into the cell media for each time point (*t* = 0 hr). The data are compiled from at least 3 independent experiments. Error bars indicate SEM.

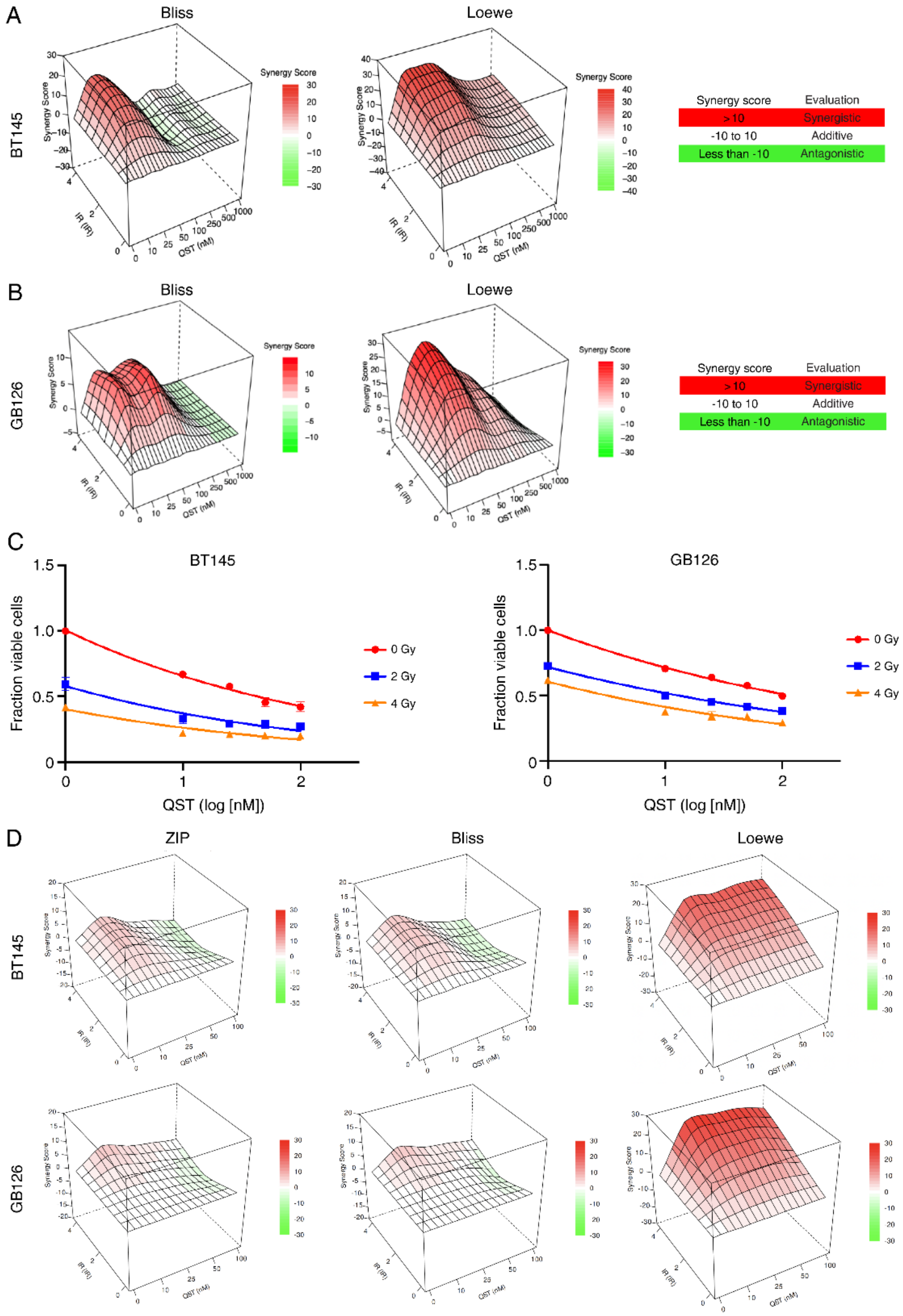

Supplemental Figure 3. QST synergizes with IR in vitro. Matrices illustrating the Bliss and Loewe synergy scores when combining QST (0–1000 nM) with increasing doses of radiation in BT145 **(A)** and GB126 **(B)**. **(C)** Dose response curves combining QST (0–100 nM) and IR treatment (0, 2, and 4 Gy) obtained by performing the sulforhodamine B assay in BT145 and GB126. For each cell line, the data are compiled from at least 3 independent experiments. Mean values are shown. Error bars indicate SEM. **(D)** Matrices illustrating the zero interaction potency, Bliss and Loewe synergy scores when combining QST with increasing doses of radiation using sulforhodamine B cell viability data as input.

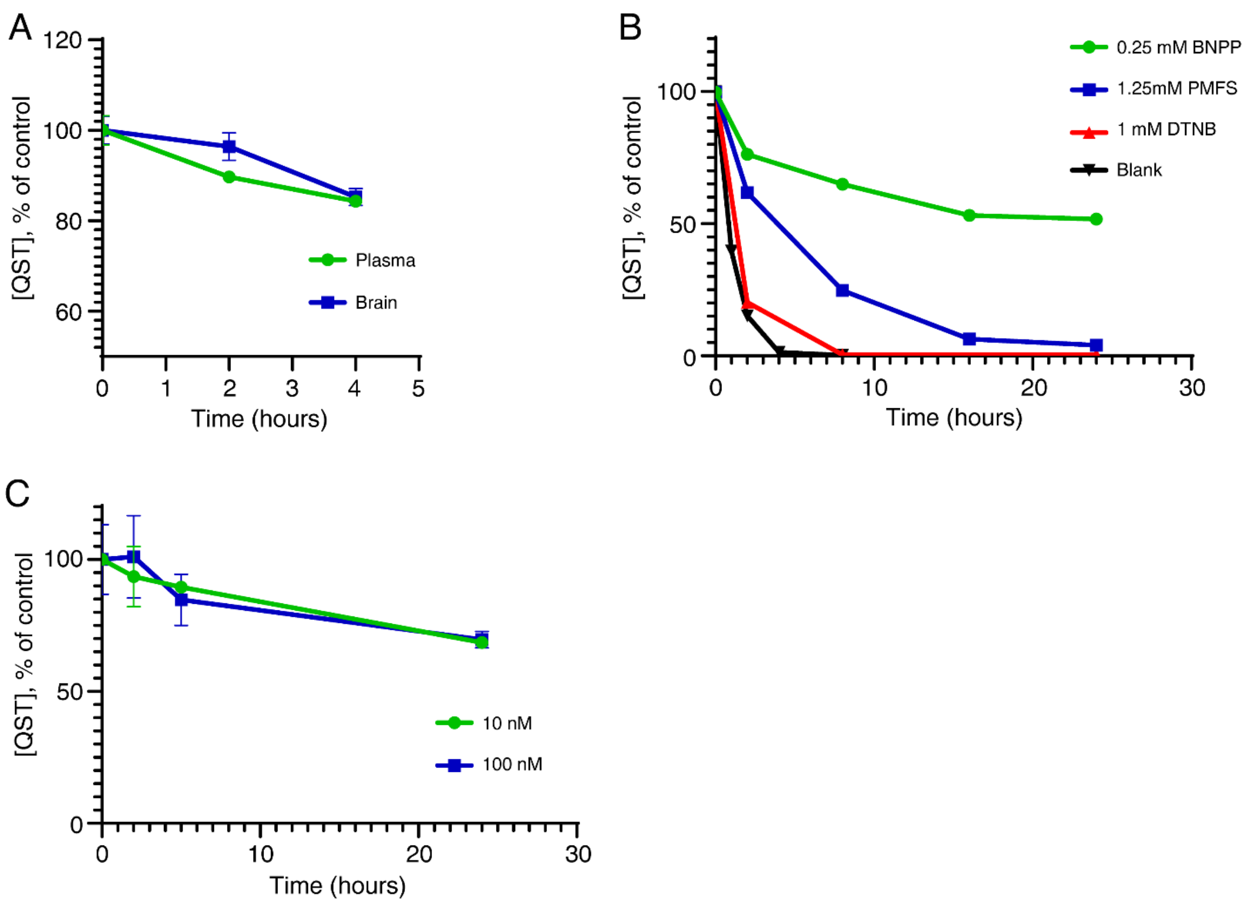

Supplemental Figure 4. Stability of QST in mouse plasma is improved upon addition of esterase inhibitors. **(A)** Stability of QST (100 nM) in mouse plasma and mouse brain homogenate when sample preparation is performed at 4 °C. **(B)** Stability of QST (500 nM) in mouse plasma spiked with 3 different esterase inhibitors: 0.25 mM bis(p-nitrophenyl) phosphate (BNPP), 1.25 mM phenylmethylsulfonyl fluoride (PMSF), and 1 mM dithiobis(2-nitrobenzoic acid) (DTNB) over the course of 24 hours at 37 °C. **(C)** Stability of QST (10 nM and 100 nM) in neurobasal cell culture medium over the course of 24 hours at 37 °C.

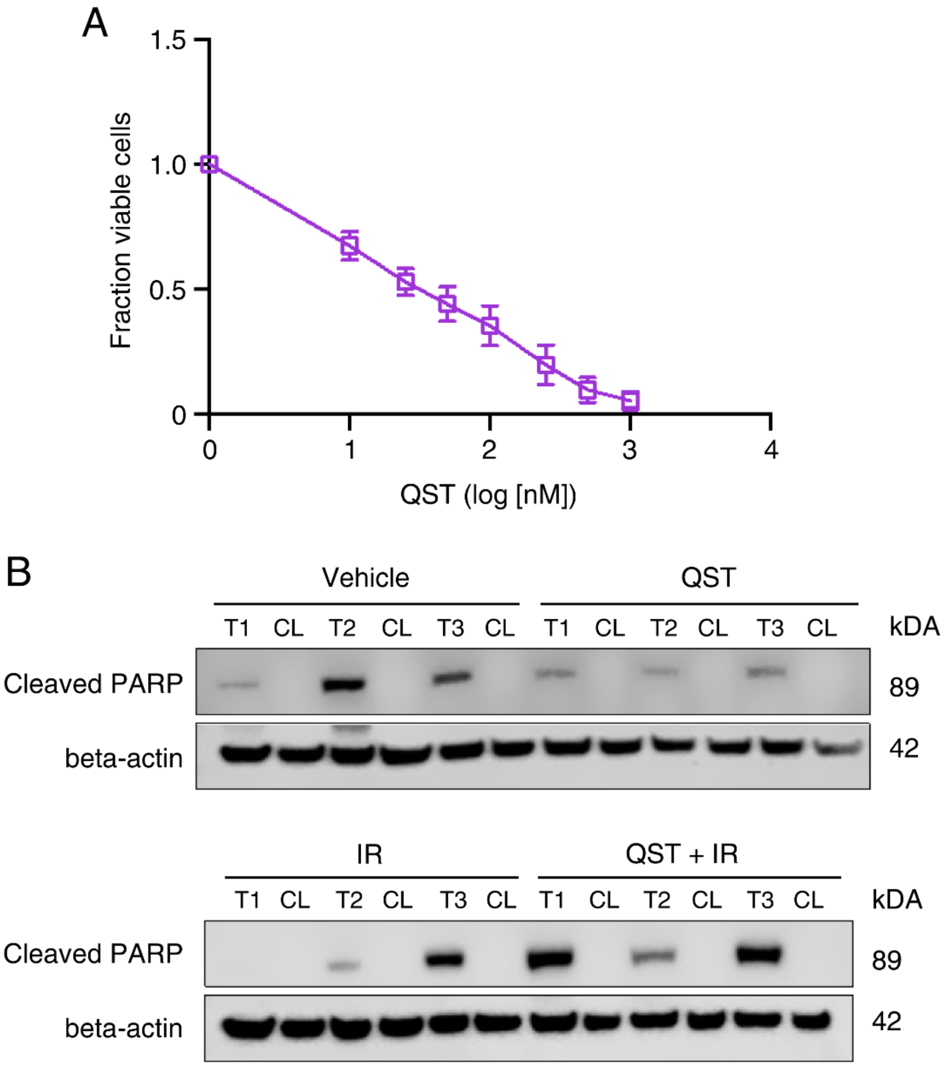

Supplemental Figure 5. QST induces cell death in tumor tissue but not surrounding normal brain tissue in vivo. **(A)** Dose-response curves with QST (10–1000 nM) in primary human astrocytes. Cell viability 3 days after treatment with QST. Mean values are shown, and error bars indicate SEM. **(B)** Immunoblotting of protein lysates derived from homogenized brain tumors and respective contralateral normal brain tissue from each cohort (n=3 mice per cohort). Membranes were probed for cleaved poly (ADP-ribose) polymerase (PARP) and B-actin. CL = contralateral normal brain tissue (opposite hemisphere); T = tumor.

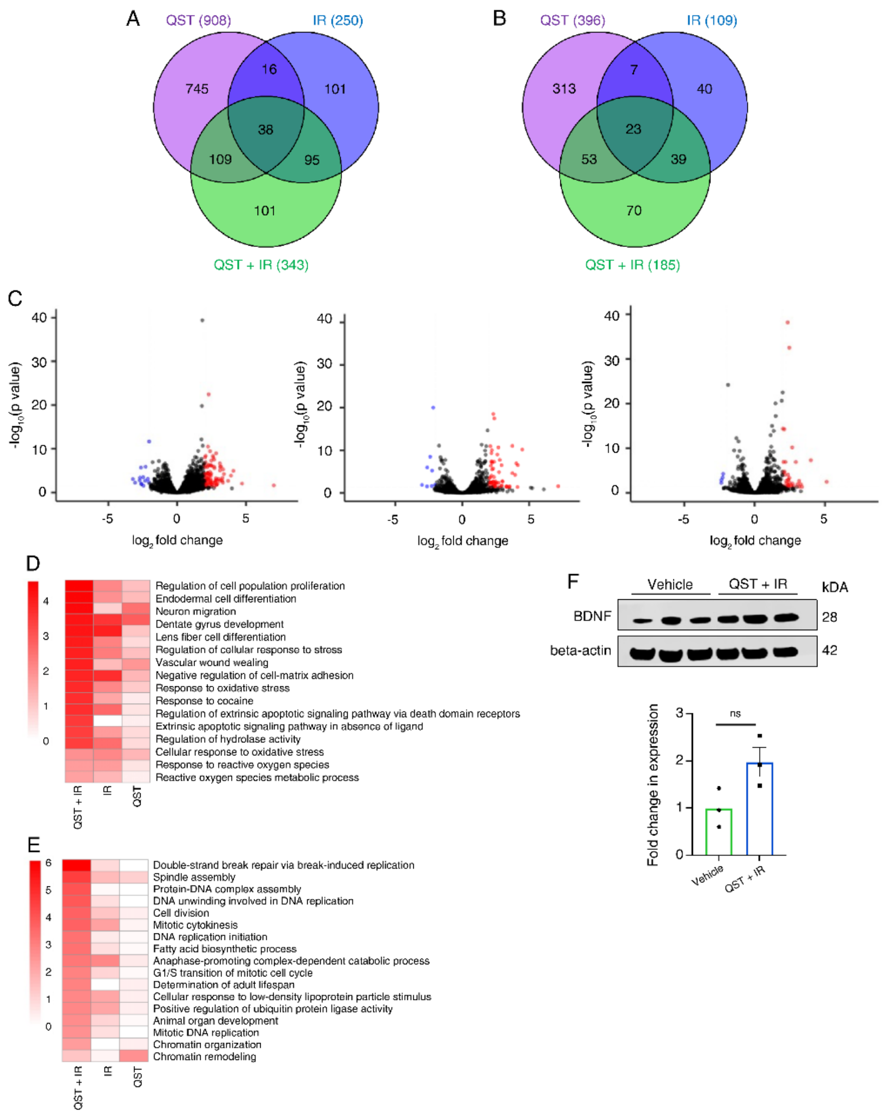

Supplemental Figure 6. RNA-seq analysis of short-term (acute) QST treatment in vivo. Venn diagrams showing the overlap in genes either upregulated **(A)** or downregulated **(B)** in response to QST monotherapy, IR treatment, or combination treatment (QST+IR). Mice received only 3 doses in total and were sacrificed 3 hours after the last dose of QST. Gene numbers in each section are shown in parentheses. **(C)** Volcano plots showing the $-\log_{10}$ (p value) and $\log_2$ fold change for transcripts detected by RNA-seq analysis of acutely treated tumors treated with QST (*left*), IR (*middle*), or QST+IR (*right*). Significantly up- and downregulated genes (false discovery rate <0.05, 2-fold) are marked in red and blue, respectively. GO analysis of genes upregulated **(D)** or downregulated **(E)** in GB126 tumors due to QST, IR, or QST+IR treatment. **(F)** Immunoblot showing increased expression of the BDNF precursor protein in whole-cell lysates from brain tumors of mice treated with vehicle or QST+IR (from moribund survival endpoint mice). Fold change in protein expression of BDNF relative to vehicle samples are shown below the immunoblot (n=3 samples per cohort). For each cohort, the data are compiled from 3 independent animal-bearing brain tumors. Circles and squares indicate values, bars indicate mean value, and error bars indicate SEM. All p values were calculated using unpaired 2-tailed *t*-test. ns = not significant.
